# Structural insights into heme binding to IL-36α proinflammatory cytokine

**DOI:** 10.1038/s41598-019-53231-0

**Published:** 2019-11-15

**Authors:** Amelie Wißbrock, Nishit B. Goradia, Amit Kumar, Ajay Abisheck Paul George, Toni Kühl, Peter Bellstedt, Ramadurai Ramachandran, Patrick Hoffmann, Kerstin Galler, Jürgen Popp, Ute Neugebauer, Kornelia Hampel, Bastian Zimmermann, Susanne Adam, Maximilian Wiendl, Gerhard Krönke, Iqbal Hamza, Stefan H. Heinemann, Silke Frey, Axel J. Hueber, Oliver Ohlenschläger, Diana Imhof

**Affiliations:** 1 Pharmaceutical Biochemistry and Bioanalytics, Pharmaceutical Institute, University of Bonn, D-53121 Bonn, Germany; 2 CS Protein Production, Leibniz Institute on Aging/Fritz Lipmann Institute, D-07745 Jena, Germany; 3 Institute of Organic and Macromolecular Chemistry (IOMC), Friedrich Schiller University Jena, D-07743 Jena, Germany; 4 Center for Sepsis Control and Care (CSCC), Jena University Hospital, D-07747 Jena, Germany; 5 Leibniz Institute of Photonic Technology (Leibniz IPHT), D-07745 Jena, Germany; 6 Institute of Physical Chemistry and Abbe Center of Photonics, Friedrich Schiller University Jena, D-07743 Jena, Germany; 7 Biaffin GmbH & Co KG, D-34132 Kassel, Germany; 8 Department of Internal Medicine 3 – Rheumatology and Immunology, University of Erlangen-Nürnberg (FAU) and University Hospital Erlangen, D-91054 Erlangen, Germany; 9 Department of Animal & Avian Sciences, University of Maryland, College Park, MD 20742 USA; 10 Department of Cell Biology & Molecular Genetics, University of Maryland, College Park, MD 20742 USA; 11 Center for Molecular Biomedicine, Department of Biophysics, Friedrich Schiller University Jena and Jena University Hospital, D-07745 Jena, Germany; 12 Present Address: European Molecular Biology Laboratory, D-22607 Hamburg, Germany

**Keywords:** Interleukins, Solution-state NMR

## Abstract

Cytokines of the interleukin (IL)-1 family regulate immune and inflammatory responses. The recently discovered IL-36 family members are involved in psoriasis, rheumatoid arthritis, and pulmonary diseases. Here, we show that IL-36α interacts with heme thereby contributing to its regulation. Based on in-depth spectroscopic analyses, we describe two heme-binding sites in IL-36α that associate with heme in a pentacoordinated fashion. Solution NMR analysis reveals structural features of IL-36α and its complex with heme. Structural investigation of a truncated IL-36α supports the notion that the N-terminus is necessary for association with its cognate receptor. Consistent with our structural studies, IL-36-mediated signal transduction was negatively regulated by heme in synovial fibroblast-like synoviocytes from rheumatoid arthritis patients. Taken together, our results provide a structural framework for heme-binding proteins and add IL-1 cytokines to the group of potentially heme-regulated proteins.

## Introduction

Regulatory heme is critical for physiological and pathological processes including neurodegenerative diseases and inflammation^1–6^. In fact, several processes such as ion channel modulation^3^ and transcriptional regulation^7^ are influenced by transient heme interactions^2^. So-called heme-binding motifs (HBM) or heme-regulatory motifs (HRM) are responsible for heme association and allow for a fast dissociation of heme due to moderate binding affinities^8–11^. Although the number of known heme-regulated proteins is increasing, structural data is rare. Detailed functional and structural analysis of a plethora of HRM-containing peptides and proteins was performed in earlier studies with a focus on Cys-based (e.g., HRI, IRP2, ALAS, CBS)^12–15^, His-based (e.g., CLOCK, Aβ, Slo1, cardiac K_ATP_ channels)^4,16–19^, and Tyr-based motifs (e.g., PGRMC1)^20^. These investigations confirmed the specific role of cysteine residues for heme binding in the context of Cys-Pro dipeptide motifs (CP motifs)^9,10^. *In silico* analysis and a database search for CP-motif-containing proteins^9^ revealed a CP-motif in interleukin (IL)-36α, a member of the IL-1 superfamily. Sequence alignment and evaluation of the IL-36 family disclosed potential HRMs in all agonistic IL-36 cytokines with the CP motif being unique for IL-36α.

The IL-1 superfamily is a group of cytokines that act as key mediators of inflammatory responses explaining their pivotal role in chronic inflammatory diseases including rheumatoid arthritis (RA) and psoriasis^21^. IL-36 cytokines, namely IL-36α, IL-36β, IL-36γ, and the natural antagonist IL-36Ra, are members of the IL-1 family, which signal through the heterodimeric receptor complex IL-36R/IL-1RacP, mediating a pathway involving the activation of NF-κB and MAP kinases. Specific N-terminal proteolytic truncation of all IL-36 family members is required to generate the active cytokine species (trIL-36)^22,23^. Ultimately, IL-36 signaling leads to the production of proinflammatory cytokines, e.g. IL-6 and IL-8^24–26^. Since all three IL-36 cytokines signal through the same receptor complex, the occurrence, the level of cytokine production, and the activation mechanisms are discussed as critical differences between the individual family members^24^. Over the past decade, a pivotal role of IL-36 cytokines in inflammatory diseases has been described, in particular in psoriasis, a chronic, multifactorial skin disease^24–27^. An anti-IL-36R antibody (ANB019, ANAPTYSBIO) for generalized pustular psoriasis (GPP) treatment is currently under investigation and has reached in-human clinical trials^28^. However, effectors and regulatory mechanisms of IL-36 cytokines in both physiological and pathophysiological conditions remain largely unknown hampering the development of specific therapies^24^.

**Figure 1:**
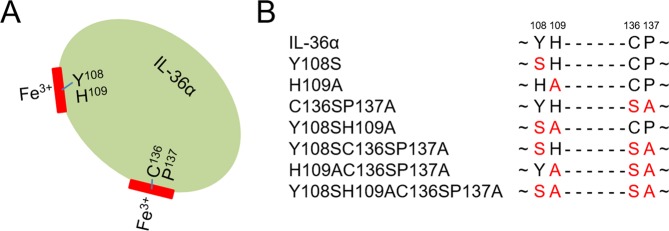
Schematic representation of IL-36α and the derived mutants used in this study. (**A**) Graphical abstract of heme binding to IL-36α. (**B**) Heme-binding site mutants of IL-36α.

We here demonstrate that IL-36-mediated signaling is significantly reduced upon heme association in human fibroblast-like synoviocytes from RA patients as detected by decreased p38 activation and diminished mRNA levels of IL-6 and IL-8. Structural analysis of the complex of heme with IL-36α and several IL-36α mutants (Fig. 1) was performed using spectroscopic methods including SPR, UV/Vis-, resonance Raman-, and 3D NMR spectroscopy. We present the binding characteristics and the solution NMR structure of IL-36α in complex with heme based on our recently published assignment of the free protein^29^. The findings presented herein extend the field of alternative heme functions by providing structural data on a potentially heme-regulated protein in complex with heme as well as revealing and classifying the network of hitherto unexploited heme effects.

## Results

### Human agonistic IL-36 family members bind heme

**Figure 2:**
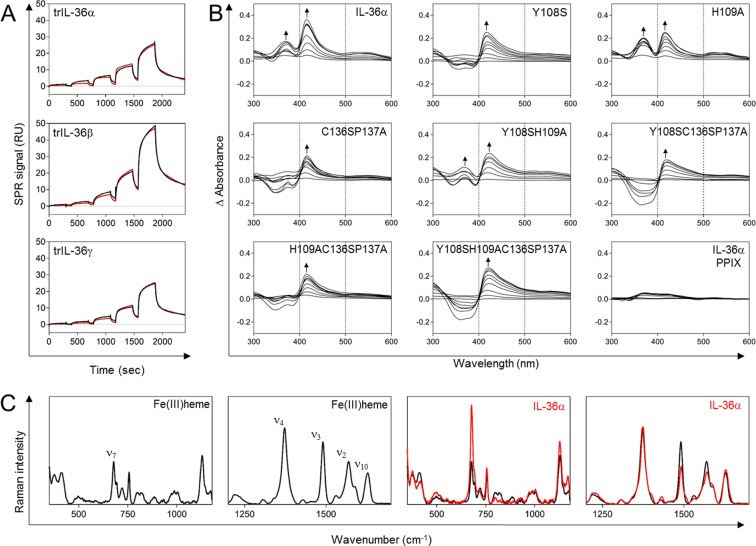
Spectroscopic studies on heme binding to agonistic IL-36 family members and IL-36α protein mutants. (**A**) SPR signal (RU) of trIL-36α, β, and γ with five consecutive heme injections of increasing heme concentrations (80 nM to 20 μM). The single-cycle kinetics method was employed (fit is displayed in red). (**B**) UV/Vis differential spectra of heme-incubated IL-36α and protein mutants. Arrows denote UV/Vis maxima whereas dashed lines at 400 and 500 nm indicate UV/Vis band width in order to illustrate band broadening which was previously found for unspecific heme binding^30^. (**C**) Raman spectra of heme (in black), and pentacoordinated wild-type IL-36α (in red) including wavenumber fingerprint region with assignment of prominent normal-mode frequencies ν_7_ (681 cm^−1^), ν_4_ (1374 cm^−1^), ν_3_ (1492 cm^−1^), ν_2_ (1571 cm^−1^), and ν_10_ (1628 cm^−1^) for heme.

There is rising evidence for tissue-dependent IL-36 expression but only little information on molecular regulation mechanisms of IL-36-mediated signaling^24^. IL-36 sequence evaluation revealed potential heme-binding sites raising the question whether heme acts as an IL-36 effector. Indeed, surface plasmon resonance (SPR) analysis revealed heme binding to the biologically active (truncated, tr) cytokines trIL-36α (aa 6–158), trIL-36β (aa 5–157), and trIL-36γ (aa 18–169, Fig. 2A). Bovine serum albumin (BSA) and lysozyme served as positive and negative controls, respectively, as reported earlier^6^ (Supplementary Fig. S1A). Sensograms of BSA and IL-36 proteins interacting with heme showed a complex biphasic binding profile. The best data fits were generated by a global heterogeneous ligand model, assuming the presence of two independent heme-binding sites. Yet, the data obtained did not disclose a distinct binding mode, such as cooperative binding of two heme molecules to one protein molecule. All trIL-36 proteins showed heme binding with comparable K_D_ values (Supplementary Table S1). A similar binding behavior was observed for IL-36α and trIL-36α, with a slightly faster ligand association to trIL-36α (Fig. 2A, Supplementary Fig. S1A). K_D_ values for both proteins were in the range of 3 to 4 µM for the interaction of the first heme molecule and 9 to 13 µM for the second binding event. In addition, the binding kinetics of the heme-IL36 interaction can be distinguished into a faster and a slower binding event.

Next, multiple sequence alignment was performed by Clustal Omega^31^ in order to evaluate potential HRMs for a 2:1 (heme:protein) binding process (Supplementary Fig. S1B, Table S2). Potential HRMs based on His, Tyr, and Cys as the axial ligands were found in all three cytokines, including both isoforms of IL-36β and γ (Supplementary Fig. S1, Table S3). Especially, in IL-36α a CP motif (SEGG**C**136**P**137LIL) and a YH motif (FLF**Y**108**H**109SQSG) were identified and further analyzed. Heme binding to the proposed 9mer CP-motif revealed a UV shift to ~366 nm with a binding affinity of 3.75 ± 0.77 µM^9^. Alanine mutants of Cys (A136P) and Pro (CA137) confirmed the essential role of Cys for binding and a reduction of binding affinity upon Pro mutation^9^ (Supplementary Fig. S1C, Table S4). For the YH-based HRM a K_D_ of 4.48 ± 2.20 µM (~415 nm) was determined. Surprisingly, mutation of His and/or Tyr, i.e. YA109 and A108H mutants, indicated heme interaction via tyrosine (Supplementary Fig. S1C, Table S4), although earlier studies revealed higher binding affinities of His-based as compared to Tyr-based motifs^11^. The same approach applied to IL-36α disclosed potential HRMs in IL-36β and γ (Supplementary Fig. S1B, Table S2), yet IL-36α was chosen for in-depth spectroscopic and structural studies due to the unique presence of the CP motif.

IL-36α and trIL-36α were obtained by overexpression in *Escherichia coli* (Supplementary Fig. S1D). Heme binding to the recombinantly produced IL-36 wild-type proteins (IL-36α, trIL-36α) was initially confirmed by SPR analysis (see above) and a fluorescence-based approach (Supplementary Fig. S1E).

To investigate the heme-binding mode in more detail, UV/Vis measurements were performed using the experimental set-up established earlier (Fig. 2B)^8,9^. BSA and lysozyme were again included as controls (Supplementary Fig. S1F). Upon heme application to IL-36α the differential spectra exhibited absorbance maxima at ~369 nm and ~416 nm. A hypsochromic UV/Vis shift to ~370 nm is characteristic for heme binding via a CP motif in a pentacoordinated fashion^9,10,32^. The second absorbance maximum at ~416 nm is indicative of an additional interaction site involving another coordinating residue. The K_D_ values of the heme-IL-36α interactions were 3.63 ± 2.67 µM (~369 nm) and 11.50 ± 3.06 µM (~416 nm). This indicated an interaction with two heme molecules per protein as suggested by SPR. No effect was observed when protoporphyrin IX (PPIX) was applied to IL-36α, supporting the essential role of the heme iron (Fig. 2B). In order to examine the relevance of the two proposed HRMs, seven IL-36α protein mutants were produced as full-length and truncated forms in *E. coli*, i.e. Y108S, H109A, Y108SH109A, C136SP137A, Y108SC136SP137A, H109AC136SP137A, and Y108SH109AC136SP137A (Supplementary Fig. S1D). The UV/Vis differential spectra obtained for the protein mutants suggested altered heme binding compared to wild-type IL-36α in all cases (Fig. 2B, Supplementary Fig. S1). Mutation of Y108 led to a significant decrease of binding affinity from 11.50 ± 3.06 µM (wild-type) to 30.69 ± 7.49 µM (Y108S) and to a loss of the UV/Vis shift to ~369 nm. The spectrum suggested a significant role of Y108 for heme binding to wild-type IL-36α. In contrast, the differential spectra of H109A was rather similar to the wild-type protein and displayed a shift to ~369 nm (K_D_ 0.77 ± 0.53 µM) and to ~416 nm (K_D_ 10.35 ± 3.16 µM), indicating that H109 has only a minor impact on heme binding. The differential spectra of the Y108SH109A mutant showed a less pronounced maximum at ~368 nm compared to wild-type IL-36α. Here determination of the binding affinity was not possible. In addition, a maximum at ~420 nm appeared (K_D_ 13.64 ± 4.69 µM). For the C136SP137A mutant a loss of the ~370 nm shift was observed suggesting that the CP motif is participating in heme binding to IL-36α (Fig. 2B), which could be further confirmed by the mutants Y108SC136SP137A, H109AC136SP137A, and Y108SH109AC136SP137A. The only exception in this context, i.e. no mutation of the CP-motif, was mutant Y108S for which no clear conclusion could be reached so far. Moreover, a UV/Vis shift to ~420 nm was found for mutant proteins Y108SC136SP137A (~419 nm, K_D_ 15.17 ± 4.35 µM), H109AC136SP137A (~417 nm, K_D_ 13.29 ± 3.12 µM), and Y108SH109AC136SP137A (~421 nm, K_D_ 14.23 ± 3.82 µM). It is worth noting that a striking broadening of the UV/Vis band at ~415–420 nm occurred in all Y108S protein mutants. This phenomenon was earlier found to be characteristic for a less specific or unspecific heme-protein interaction^30^. Therefore, it can be hypothesized that the Y108 mutation in IL-36α results in a loss of heme-binding specificity. Taking the peptide-based measurements into consideration, these observations indicate that the YH motif serves as heme ligand with Y108 representing the coordinating residue, and H109 supporting heme association. Thus, involvement of the suggested HRMs (C136P137, Y108H109) was successfully verified with different protein mutants. It was recognized, however, that additional residues might be involved in heme binding, yet in an unspecific fashion. In order to gain further insight into the complex geometry resonance Raman spectroscopy was applied.

### Raman spectroscopy revealed a pentacoordinated IL-36α-heme complex

Resonance Raman (rRaman) spectroscopy with an excitation wavelength of 413 nm was conducted to analyze the heme-iron coordination state by selective enhancement of the characteristic vibrations of the heme moiety as previously established (Fig. 2C, Supplementary Fig. S2, Tables S1, S4)^10,11,33–35^. Wild-type IL-36α predominantly forms a pentacoordinated heme-complex as the coordination marker band ν_3_ is still found around 1492 cm^−1^, despite a neglectable shoulder around 1508 cm^−1^ and a slightly increased intensity of the ν_7_ band (Fig. 2C). The H109A mutant shows pentacoordination and of all variants the highest similarity with the wild-type IL36α-heme complex (Supplementary Fig. S2A). In agreement with the UV/Vis studies, the loss of H109 has the least effect on heme binding. Y108S mutation (also in Y108SH109A) diminished the ν_2_ and the ν_3_ bands reduce, which further supports heme coordination via Y108. This is in accordance with the UV/Vis and rRaman data of the corresponding peptide- and protein-heme complexes (Supplementary Fig. S2, Tables S1, S4).

Mutations in the second heme-binding site, i.e. C136SP137A, led to the formation of a weak shoulder around 1504 cm^−1^ at the long wavelength side of the coordination-state sensitive ν_3_ band (Supplementary Fig. S2A, Table S1).

### Full-length IL-36α and truncated IL-36α bind heme in a similar manner

**Figure 3:**
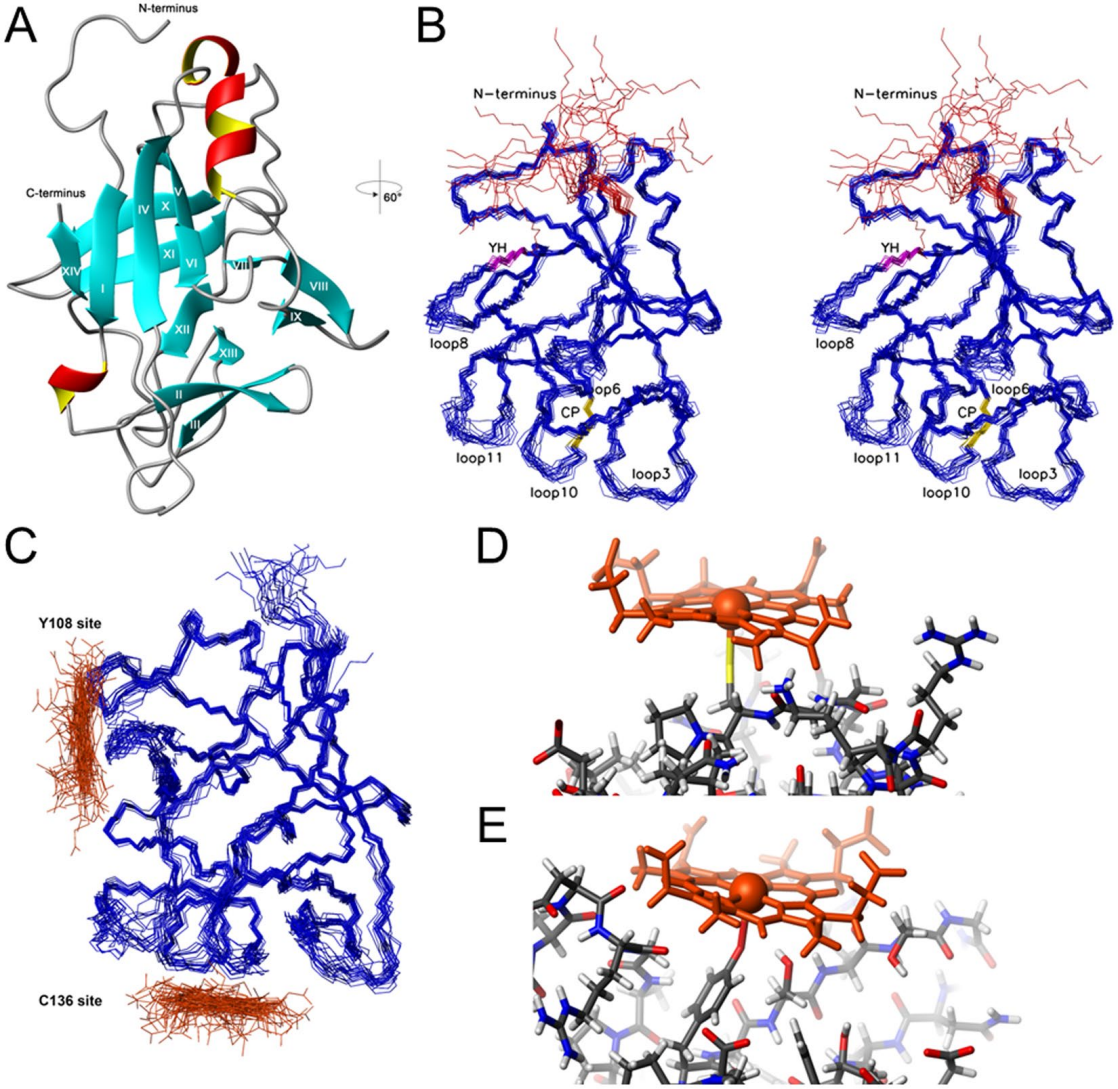
Structural analysis of IL-36α. (**A**) NMR structure of IL-36α with numbered β-sheet elements indicated by cyan arrows, α-helical elements in red/yellow and loop regions in grey. (**B**) Stereoview of the 20 best energy minimized conformers. Flexible loops are indicated and the flexible N-terminal residues, the C136P137, and Y108H109 sites are colored (Met1-Thr9 – red, Cys136Pro137 – gold, Tyr108His109 – magenta). (**C–E**) Bundle of the IL-36α-heme complex (IL-36α backbone in blue, heme in orange) (**C**). Detailed view of the heme coordination at Cys136 (**D**) and Tyr108 (**E**).

**Figure 4:**
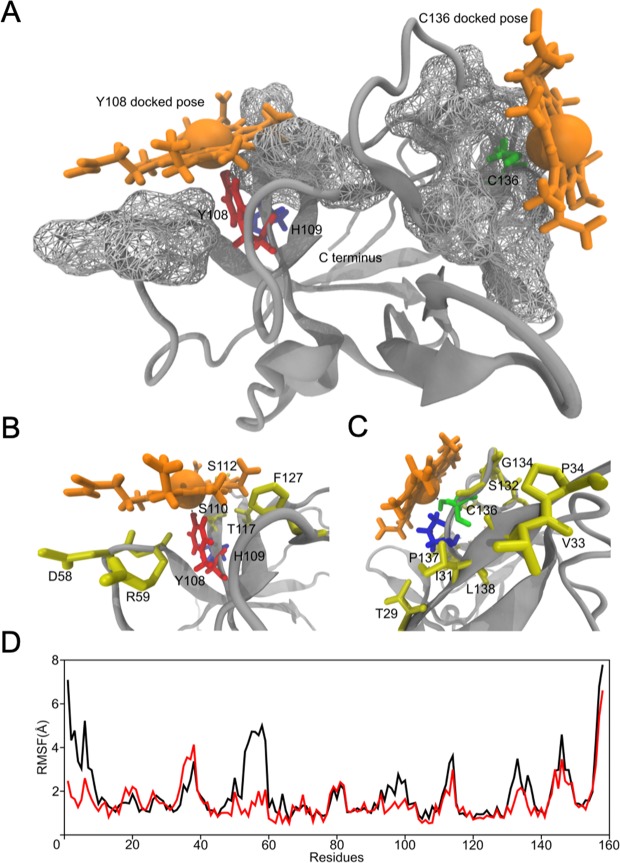
Heme binding to IL-36α and its effects on the protein supported by molecular docking and MD simulation studies. (**A–C**) The structure of wild-type IL-36α (grey) with two heme molecules (orange) docked at C136 (green) and Y108 (red), respectively (H109, blue). Wireframe surfaces drawn around both binding sites (C136, Y108) represent surfaces of IL-36α residues that make contact with the heme molecule as predicted by the docking algorithm (**A**). A zoom in at Y108 (**B**, Y108, red; H109, blue) and C136 (**C**, C136, green; P137, blue). Residues that stabilize the heme-bound conformation (yellow) are labeled. (**D**) Comparative per-residue root mean squared fluctuation (RMSF) profiles of the heme bound (red) and the free states (black) of the protein. The RMSF profiles were generated from 200 ns equilibration MD simulations of the free protein and the protein-heme complex. Clear reduction of fluctuations from their mean position is noticed for several amino acids in the heme-bound state indicating the effect of heme binding to the protein.

To determine the solution structure of IL-36α we performed heteronuclear 3D NMR spectroscopy on the basis of previously derived NMR resonance assignments^29^. The conformers were calculated based on 4107 NOE constraints (Supplementary Table S5). The structure of IL-36α (Fig. 3A,B) consists of 14 β-strands in three different β-sheets (XIV-I-IV-V-VI-VII-X-XI-XII-XIII, II-III, VIII-IX) composed in a canonical β-trefoil fold^36^ as typical for cytokines of the IL-1 family^37^. These are supplemented by one short α-helix (I92-N97) of approximately 1.5 turns and two short 3_10_-helical elements composed of three residues each. Five of the eleven loops (P34-M39 (loop 3), K79-Q82 (loop 6), S112-N115 (loop 8), E133-G135 (loop 10), L144-A147 (loop 11); (Fig. 4B) connecting the different β-strands exhibit higher structural disorder due to increased dynamics as also supported by the heteronuclear NOE data. This also includes loop 10 between strands VII and VIII of β-sheet 1, which directly precedes one of the potential heme-binding residues: Cys136. Superimposition with the X-ray structure of IL-36γ (sequence homology 67% at 57% identity; PDB code 4IZE; Supplementary Fig. S6) results in a root-mean-square deviation (r.m.s.d.) of 1.63 Å for the backbone atoms of the ordered stretch P10-F158 excluding the loops.

The {^15^N,^1^H}-heteronuclear NOE data demonstrate that IL-36α with exception of the first ten N-terminal residues behaves as a rigid molecule (Supplementary Fig. S3B). CD analysis confirmed the secondary structure elements of IL-36α obtained from the NMR studies by revealing a content of β-sheet fold between 20 and 35% (Supplementary Table S6, Fig. S3A). Comparison of the [^1^H,^15^N]-HSQC spectra of IL-36α and trIL-36α (Supplementary Fig. S3C) revealed - with exception of the missing cross peaks for the cleaved five amino acids - no major changes in the NMR fingerprint, thereby indicating that the truncation did not affect the 3D structural fold of the protein. In addition, the suggested HRMs (Y108H109, C136P137) are located at the C-terminus, while sequence truncation occurs at the N-terminus. Therefore, no significant change of the heme-binding behavior was expected. As aforementioned, binding studies with both proteins incubated with heme verified that heme binding occurred in a similar manner (Fig. 2A, Supplementary Fig. S1).

The NMR spectra of the IL-36α-heme complex indicated no change in the structural scaffold upon heme binding. In addition to the NMR experiments with Fe(III)-heme, SPR and NMR measurements were performed with Ga(III)-protoporphyrin as previously used^10,11,38,39^. The data suggested a similar interaction as observed for Fe(III)-heme. Variations in [^1^H,^15^N]-HSQC NMR cross peak intensities upon addition of heme to IL-36α (100 µM) were used to map the interaction interface. The superposition of the [^1^H,^15^N]-HSQC spectra of trIL-36α in the free and heme-bound state revealed a decrease in signal intensities for residues N19, R21, V22, I44, H51, T54, N61, Y108, H109, G113, R114, C136, D151, F152 (Supplementary Fig. S3D). Thus, the NMR data of IL-36α-heme suggested that the CP motif functions as coordination site (Fig. 3C–E). Moreover, the spectrum showed that at the given threshold the signal of H109 vanishes as a consequence of heme coordination to Y108, thereby supporting the attributed function of the YH site as second binding platform for heme besides C136 (Supplementary Fig. 2C–E).

Based on the NMR solution structure of IL-36α, we studied the possible binding mode of IL-36α and IL-36α-heme to its cognate receptor. Employing the coordinates of the ternary complex interleukin-1 receptor type-2 (IL-1RII)/interleukin-1 receptor accessory protein (IL-1RAcP)/interleukin-1β (IL-1β; PDB 3O4O)^40^, we first superimposed the IL-36α structure onto the IL-1β moiety in the complex (cf. Supplementary Fig. S3E for sequence alignment). The r.m.s.d. of 2.15 Å (IL-36α residues 10–15, 20–28, 29–34, 42–49, 60–87, 101–143, 149–158) indicates a good agreement of the backbone folds when neglecting loop areas (Supplementary Fig. S6). The sequence mismatch between IL-1β and residues around K57-D58-R59 in IL-36α leads to a shortened loop conformation and thereby to a steric decompression of the molecular interaction area. Superposition revealed two major steric clashes (cf. Supplementary Fig. S6A,C). Although flexible, the N-terminal residues M1-L5 cannot be accommodated in the V-shaped cleft between two β-sheet elements near residues W260 and G280 of IL-1RII. However, binding of truncated trIL-36α is not hindered because the remaining flexible amino acids K6-T9 are positioned in the upper and opened part of this V-shape. This might explain why N-terminal processing of IL-36α is required for biological activity. In the superposition the flexible loop K36-M39 clashes with the stretch H21-R24 of receptor domain-I. However, domain-I is linked to receptor domain-II by a short bent linker element (E114-T116). A rotation centered at this linker element allows for repositioning domain-I to avoid disruptive van der Waals interactions between the receptor and IL-36α. The X-ray structure of the complex reveals that domain-I of the IL-1RII displays only one stabilizing hydrogen bond to domain-II (R27Hη12/22-Y125Oη), which would be perished by the domain reorientation. In contrast, no major domain movement is necessary to accommodate IL-36α at domain-III. Heme binding to trIL-36α before complex formation increases the gross shape of the molecule and leads to further steric impacts especially in the linker region between domains II and III of IL-1RAcP which may diminish the functional efficacy of the receptor complex.

### Computational studies support experimental results for heme binding to IL-36α

The NMR solution structures of free and heme-bound IL-36α facilitated investigations of heme binding to the protein and *in silico* structure modelling of the mutant proteins by employing molecular docking and molecular dynamics (MD) simulations (Fig. 4, Supplementary Fig. S4). Heme binding predicted by the Vina^41^ docking algorithm helped to determine optimal conformations of the heme-protein interactions. Heme coordination to wild-type IL-36α was found to occur via C136 and Y108 according to the top two complexes from the docking run, which is in agreement with the experimental data (Fig. 4, Supplementary Movies S1, S2). The docked pose for binding via C136 was established by the heme iron placed at a proximity of 2.72 Å from the sulfur atom of C136. The docked pose was further characterized by stabilizing interactions of the surrounding residues namely T29, I31, A32, V33, P34, S132, E133, G134, G135, P137, L138, I139, and N148 (Fig. 4A,B). The docked pose of heme bound via Y108 had the iron ion placed 2.32 Å from the oxygen atom of Y108. This pose was supported by additional surrounding residues, namely D58, R59, H109, S110, S112, T117, and F127 (Fig. 4, Supplementary Movies S1, S2).

### Heme negatively regulates IL-36-mediated signal transduction in primary human patient fibroblasts

IL-36α induces expression of proinflammatory mediators such as IL-6 and IL-8 in synovial fibroblast-like synoviocytes (FLS) from RA patients^25^. Since heme binds to IL-36α, the question arises whether this interaction has an effect on IL-36α-induced activation of FLS. Human FLS were thus treated for 24 hours with different ratios of IL-36α in complex with heme.

**Figure 5:**
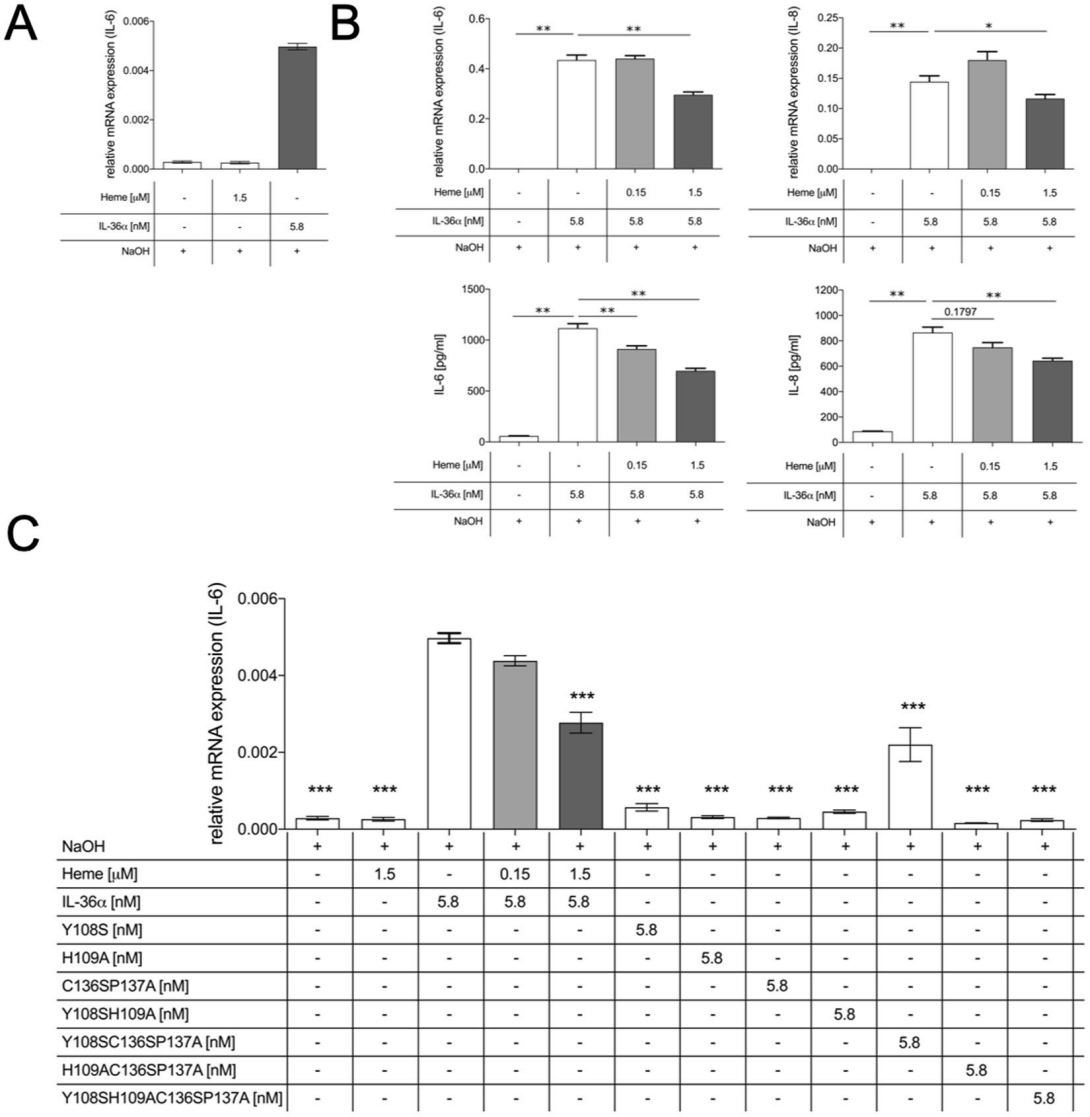
Heme impairs IL-36-mediated cytokine production in human patient FLS. FLS from RA patients treated with heme (**A**) or two ratios of heme-IL-36α (**B**) for 24 h. Relative mRNA expression of IL-6 and IL-8, normalized to GAPDH, analyzed by quantitative real time PCR (qRT-PCR; top), and cytokine concentrations in the cell culture supernatant measured by ELISA (bottom). Values are means + SEM. One representative experiment (n = 6) of two independent experiments is shown. Statistical analysis was performed using Mann-Whitney Test Bonferroni corrected with *p < 0.05 and **p < 0.01. (**C**) FLS from RA patients treated with varying ratios of IL-36α or IL-36α mutants to heme for 24 h (light and dark grey bars). Relative mRNA expression of IL-6, normalized to GAPDH, and analyzed by qRT-PCR. Values are means ± SEM of one experiment (n = 3). Statistical analysis was performed using One-Way Anova Test Dunnet corrected, with *p < 0.05, **p < 0.01, and ***p < 0.0001 for comparisons with recombinant trIL-36α.

Cytotoxicity was determined to exclude that the components trigger a significant reduction of cell viability (Supplementary Fig. S5A). IL-36α (5.8 nM) alone induced IL-6 and IL-8 expression in FLS, whereas heme did not have an effect on the expression. Stimulation with the IL-36α-heme complex at two ratios (26:1 and 260:1) corresponding to 0.15 and 1.5 µM of heme resulted in significantly decreased expression of IL-6 and IL-8 compared to the controls (Fig. 5A,B). The heme concentration was chosen according to earlier studies considering the scavenging effect of unspecific heme binding to BSA^42^. Further, the treatment with IL-36α-heme led to a significant reduction of IL-6 and IL-8 release in the supernatant (Fig. 5B, lower panels). Finally, heme impaired IL-36α-mediated signaling in FLS, showing decreased activation of p38 (Supplementary Fig. S5B,C). Similar results were observed for IL-36 family members IL-36β and γ (Supplementary Fig. S5D). This confirms heme binding to all agonistic IL-36 family members as shown by SPR. In summary, the IL-36-heme interaction led to a reduced expression of IL-6 and IL-8 in human patient-derived FLS.

**Figure 6:**
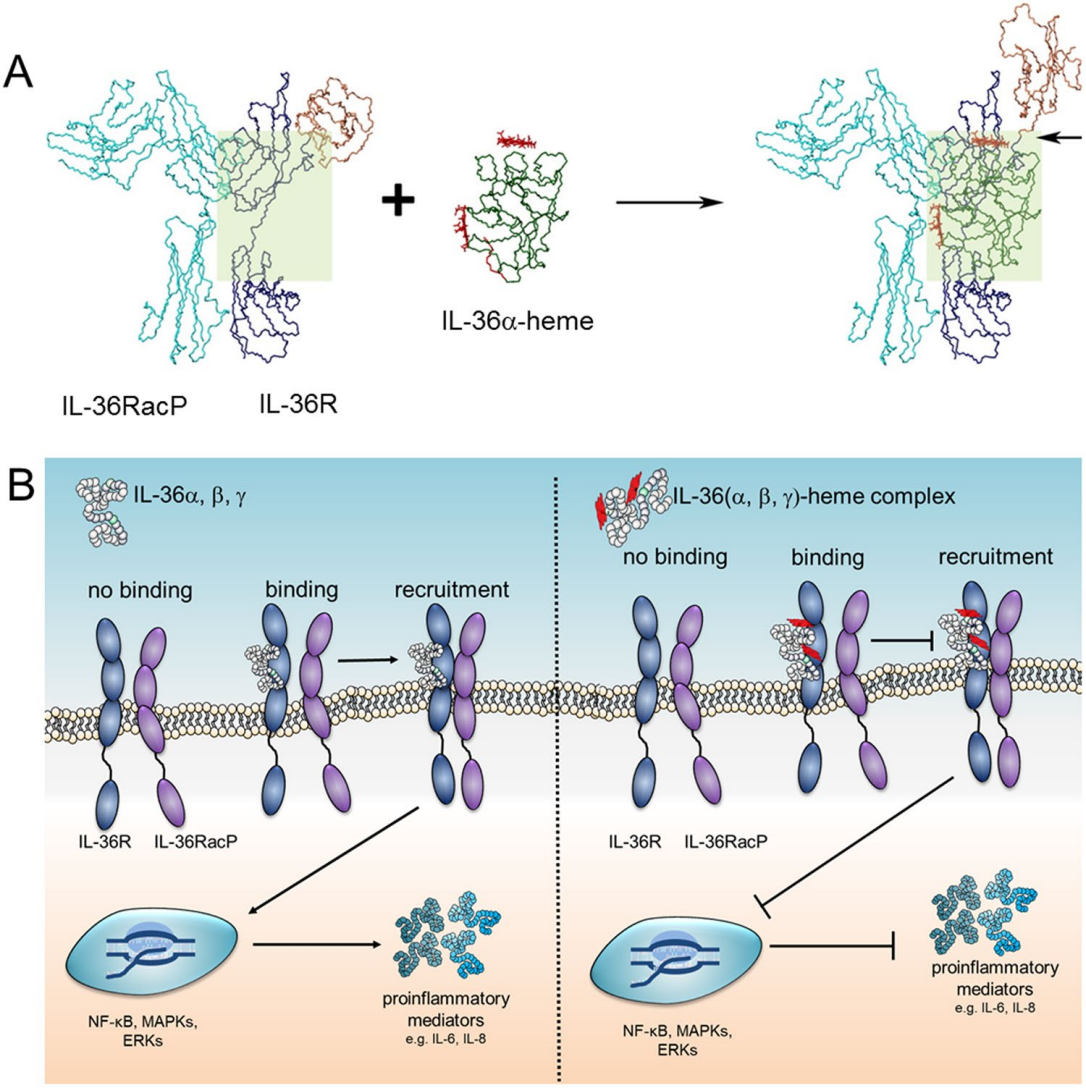
Potential physiological scenarios of IL-36-heme complex formation. (**A**) IL-36α-heme binding to the receptor IL-36R may lead to steric clashes (indicated by an arrow) as identified by docking of the complex into the X-ray structure of the IL-1RII receptor complex (PDB: 3040,^40^). (**B**) Potential scenarios explaining decreased IL-36-mediated signaling upon heme binding, either ligand binding is completely diminished or recruitment of the accessory protein (IL-36RacP) is impaired.

The IL-36α mutants were investigated using the same experimental set up to test the impact of the individual heme-binding motifs. IL-6 expression was significantly reduced in all IL-36α-mutant-treated FLS compared to IL-36α (Fig. 6C). Abrogation of IL-6 expression suggests that mutations in the heme-binding motifs of IL-36α impact the protein’s functionality. On the one hand, the mutations may have a major impact on protein folding, structure, and consequently biological activity. On the other hand, the mutated residues might be pivotal for receptor binding. In support of this, the only protein mutant with a non-mutated YH motif (mutant C136SP137A) showed biological activity, even if reduced with respect to wild-type IL-36α. This suggests a crucial role of the YH motif and surrounding amino acids for the IL-36α receptor interplay.

### IL-36α-heme complex does not exhibit peroxidase-like activity

A peroxidase-like activity of heme-peptide/protein complexes has been discussed to be critical due to cytotoxic ROS formation^4,43–45^. We thus tested the peroxidase-like activity of IL-36α-heme-complexes in two different ratios (1:1 and 1:2, protein:heme) as well as of both heme-binding motifs (CP, YH) on the peptide level (Supplementary Fig. S5E). No significant increase of the heme’s peroxidase activity (100%) was observed upon complex formation with proteins and peptide, respectively.

## Discussion

We show that heme interacts with the agonistic IL-36 family members α, β, and γ. Heme-IL-36 complex formation impairs IL-36 signaling and leads to a decreased production of the proinflammatory cytokines IL-6 and IL-8 *in vitro* (Fig. 6). Further studies are required to clarify the *in vivo* situation. It is worth noting that in case of severe hemolysis concentrations of unbound heme in the range of 20 to 350 μM have been described^46–48^.

Two heme molecules bind to one protein molecule in *in vitro* studies, one with a higher and the second with a lower binding affinity. Based on experimental and computational studies, there is strong evidence that the proposed C136P137 motif is responsible for the association with one heme molecule. For the second binding event a significant role of Y108 as heme axial ligand is proposed, while H109 acts as an assisting residue for the neighboring tyrosine. A closer look revealed that wild-type IL-36α forms a pentacoordinated complex with heme as demonstrated by *r*Raman and 3D NMR spectroscopy. According to earlier studies, CP motifs strongly tend to form pentacoordinated complexes, supporting heme binding to the C136P137 motif. Predominantly pentacoordination was also found for Y-based HRMs, supporting that the second heme is bound in a pentacoordinated fashion to Y108H109 in IL-36α, too.

Structural investigation of full-length and trIL-36α revealed that there is no major structural change upon N-terminal cleavage that is required for full biological activity. This supports the notion that the significantly reduced activity of full-length IL-36α is due to a very flexible region at the N-terminus that blocks necessary association sites for IL-36α-receptor binding. Indeed, modeling of the NMR structure of IL-36α into the X-ray structure of the receptor complex (Fig. 6A, Supplementary Fig. S6) revealed steric clashes with the IL-1RII domain-III that can be minimized by the N-terminal truncation. Heme binding to IL-36α further increases the steric demand in areas close to the IL-1RII domain-I and the linker between IL-1RAcP domains-II and -III (Fig. 6A). Whether the IL-36α-heme complex is not able to interact with the receptor IL-36R at all, or IL-1RacP recruitment is inhibited by the complex remains to be clarified (Fig. 6B). However, it was shown that the interaction between heme and IL-36α leads to a decreased IL-36-mediated signal transduction *in vitro* and would consequently evoke an anti-inflammatory effect in the organism. Except for the C136SP137A mutant, none of the other IL-36α variants exhibited biological activity impeding a functional investigation of the role of the two binding sites. The Y108H109 motif, however, appears to be of importance with regard to the protein’s functionality. In fact, point mutations in IL-36 cytokines were shown to have a significant impact on the protein function, including an alteration of agonistic/antagonistic properties as well as loss of function^37^.

In the context of pathophysiological functions of heme^1^, our findings seem to be ambiguous. However, heme has been shown earlier to inhibit the classical complement pathway mediated by C-reactive protein and immune complexes via interaction with C1q^49^ as an endogenous negative-feedback regulator. With respect to cellular damage where heme is released in high concentration, heme was suggested to support an anti-inflammatory event as part of a rescue system^49^. The role of IL-36 cytokines in such high heme-releasing conditions (e.g. trauma, malaria) is yet unclear. In addition, contributions of other interleukins in such scenarios are unknown so far. A sequence analysis of e.g. IL-1 family members and other interleukins revealed potential HRMs, too. CP motifs as such are present, for example, in IL-1Ra and IL-33. In addition, other motifs may also be suitable, e.g. a sequence stretch around H54 in IL-1β or H76 in IL-33, while heme binding to other interleukins such as IL-18 seem less likely considering an in-house evaluation for potential HRMs^71^.

To the best of our knowledge, this study is the first report on a direct heme-interleukin interaction, a comprehensive structural investigation of IL-36α, its complex formation with heme as a potential physiological regulator, and biological consequences resulting from it. This adds further human proteins to the list of possible candidates regulated by heme and increases knowledge in the field of “hemeostasis”. Furthermore, our study provides a deeper insight into structural reasons for the lack of biological activity of the full-length IL-36 protein due to disabled interaction with the receptor. We thus anticipate that the emerging IL-36 research will gain momentum, in particular with regard to the biological relevance of IL-36 regulation in physiological and pathophysiological conditions.

## Methods Details

### Recombinant protein expression

pET-28a (Novagen, Madison, Wisconsin, USA) was used as expression vector of the gene encoding 158 amino acids (long version) and 153 amino acids (truncated version, tr) of IL-36α and mutants, respectively, and was inserted into the NdeI/XhoI site. An N-terminal protein His_6_ tag and the essential thrombin cleavage site between the tag and the gene-encoding sequence were introduced into the long versions. The procedure used leaves three additional amino acids (Gly-Ser-His) attached at the N-terminus of full-length IL-36α (161 amino acids). In contrast, a caspase-3 (DEVD) cleavage site was cloned into the vector before amino acid K5 of IL-36α in the truncated proteins. Following the BL21(DE3) *E. coli* (Novagen, Madison, Wisconsin, USA) transformation, cells were plated onto kanamycin including plates. Eventually a single colony was inoculated into kanamycin containing (50 μg/mL) Luria Bertani (LB) medium. After the colonies were grown up to an OD_600nm_ 0.7, the bacteria cultures were enlarged to 500 mL of LB medium. For NMR studies of wild-type full-length IL-36α and trIL-36α, the primary cultures were inoculated into ^15^NH_4_Cl and ^13^C_6_-glucose containing M9 medium (500 mL). Protein expression was induced by 0.3 mM IPTG for 18 h at 18 °C. Subsequently a lysis buffer (50 mM Tris/HCl, 300 mM NaCl, 5 mM imidazole, 5 mM β-mercaptoethanol (pH 8)) was applied for cell lysis followed by French press and centrifuged at 10.000 × g. After lysis Ni-NTA agarose resin (GE Healthcare, Freiburg, Germany) was used for purification of the cell lysate. Upon washing with 5 mM and 10 mM imidazole-containing lysis buffer (10 column volumes each), the protein was eluted applying 0.25 M imidazole. Cleavage of the His_6_ tag was performed at 4 °C overnight using 5 U/mg of thrombin (Sigma-Aldrich, Taufkirchen, Germany). In case of the truncated proteins, 1 mg of caspase-3 (produced in-house) was used to cleave 40 mg of trIL-36α overnight in dialysis buffer (50 mM HEPES, pH 7.4, 75 mM NaCl, 2 mM DTT) at 4 °C. This procedure leads to no additional overhang residues at the N-terminus. The overnight-cleaved protein was applied on a pre-equilibrated Ni-NTA column with overnight dialysis buffer, followed by elution with same dialysis buffer to collect remaining digested protein and leaving undigested protein bound to the column. Collected flow through was concentrated to 1–1.5 mL using a 3-kDa Amicon filter before injection onto a 16/60 Hiload S75 size exclusion chromatography column (GE Healthcare, Freiburg, Germany) that was pre-equilibrated with 20 mM Tris/HCl (full-length) or 20 mM HEPES (truncated), pH 7.4, 150 mM NaCl, 2 mM DTT. Protein fractions were combined, concentrated and the buffer was replaced to 20 mM sodium phosphate buffer (pH 6.9). Samples were either lyophilized or directly used for the further experiments.

### SDS-PAGE

SDS-PAGE (gel electrophoresis) of the recombinantly expressed proteins was performed on tricine gels. 18% acrylamide/bis(acrylamide) with 5% cross-linking were used for the resolving gel, whereas the stacking gel contained 5% acrylamide/bis(acrylamide) with 3.3% cross-linking. Upon fixation with 5% glutaraldehyde (30 min) the gels were stained with colloidal Coomassie^50,51^.

### Peptide synthesis and purification

An EPS 221 peptide synthesizer (Intavis Bioanalytical Instruments AG, Cologne, Germany) was used for the synthesis of all presented peptides (**1–7**). using a standard Fmoc (N-(9-fluorenyl)methoxycarbonyl) protocol for automated solid-phase peptide synthesis and employing HBTU and HOBt as coupling reagents. Rink amide MBHA resin (0.53 mmol/g) served as polymer support. Peptide cleavage was performed adding 100 µL of reagent K (75 mg phenol, 25 μL ethandithiol, 50 μL thioanisol, 50 μL water) in 1 mL of TFA per 100 mg resin. Crude products were purified by semi-preparative RP-HPLC on a Shimadzu LC-8A system equipped with a Knauer Eurospher 100 column (C18, 250 × 32 mm, 5 μm particle size, 100 Å pore size) using gradient elution with 0.1% TFA in water (eluent A) and 0.1% TFA in 90% acetonitrile/water (eluent B) as the mobile phases. Analytical HPLC on a Shimadzu LC-10AT system with a Vydac 218TP column (C18, 4.6 × 25 mm, 5 μm particle size, 300 Å pore size) was used to analyze peptide purity. The mobile phase consisted of 0.1% TFA in 100% water as eluent A and 0.1% TFA in acetonitrile as eluent B. Applied gradients for the individual peptides are listed in Supplementary Table S3. Detection was at 220 nm in all cases^9–11^.

### Mass spectrometry

Molar masses of all peptides were detected with an LC-ESI micrOTOF-Q III system (Bruker Daltonics GmbH, Bremen, Germany) coupled to a Dionex Ultimate 3000 LC (ThermoFisher Scientific, Dreieich, Germany). An EC 100/2 Nucleoshell RP18 column (C18, 100 × 2 mm, 2.7 μm particle size, 90 Å pore size, Macherey-Nagel, Düren, Germany) was used prior to infection into the MS instrument. Mass spectra were analyzed using the Data Analysis 4.1 software (Bruker Daltonics GmbH, Bremen, Germany). Information about the molar mass of individual peptides can be found in the Supplementary Table S3.

### Amino acid analysis

Peptide content was determined by amino acid analysis applying an ion exchange chromatography system. Peptide hydrolysates (6 N HCl at 110 °C for 24 h) were analyzed using an Eppendorf Amino Acid Analyzer LC 3000. An external standard (Laborservice Onken GmbH, Gründau, Germany) was used for data evaluation. Expected results were obtained for all peptides with respect to amino acid composition. Peptide content (in %) was used to calculated concentrations of all peptides prepared and stored as lyophilized powders prior to the experiments.

### Preparation of heme solution

If not stated otherwise, Fe(III)heme referred to as heme (1 mM) was dissolved in 30 mM NaOH and incubated for 30 minutes under the exclusion of light. The solution was further diluted in the buffer system required according to the respective experiment.

### Surface plasmon resonance measurements

SPR measurements were performed on a Biacore T200 instrument (GE Healthcare Europe GmbH, Freiburg, Germany) at 25 °C. The running buffer was 10 mM HEPES (pH 7.4), 150 mM NaCl, 0.05% Tween 20. The proteins BSA (VWR Life Science AMRESCO, Germany), lysozyme (AppliChem GmbH, Darmstadt, Germany), IL-36α (protein expression), trIL-36α (R&D Systems, Minneapolis, MN, USA), trIL-36β (isoform 2, R&D Systems, Minneapolis, MN, USA) and trIL-36γ (R&D Systems, Minneapolis, MN, USA) were covalently immobilized by amine coupling on a CM5 sensor chip (GE Healthcare). Briefly, lysozyme was diluted to a final concentration of 2.5 µg/mL in acetate buffer (pH 7.0), and BSA was diluted to 2.5 µg/mL in acetate buffer (pH 5.0). trIL-36α, trIL-36β, and trIL-36γ were diluted to 0.85 µg/mL in acetate buffer (pH 4.0). Subsequently, the proteins were separately injected at 10 µL/min on an EDC/NHS activated flow cell until immobilization levels of 1600 RU (lysozyme), 1050 RU (trIL-36α), 2900 RU (BSA), 937 RU (trIL-36β), and 320 RU (trIL-36γ) were achieved. An activated/deactivated flow cell was used for reference subtraction. For determination of K_D_ values and kinetic parameters, a titration series of five consecutive injections with increasing heme concentrations (0.08 µM, 0.31 µM, 1.25 µM, 5 µM, 20 µM, diluted in running buffer) was performed at a flow rate of 30 µL/min using a standard single-cycle kinetics method implemented in the Biacore T200 Control Software (GE Healthcare). Afterwards, the surface was regenerated by two injections of 25 mM NaOH/500 mM NaCl. An injection series of running buffer for double referencing was subtracted from each curve. Data were globally fitted using the heterogeneous ligand analysis model.

### UV/Vis spectroscopy

A Multiskan GO microplate spectrophotometer (ThermoFisher Scientific, Dreieich, Germany) was used for UV/Vis measurements. Heme and peptide solutions were prepared in 100 mM Hepes buffer (pH 7.0). IL-36 cytokines (~5 µM), dissolved in 20 mM sodium phosphate buffer (pH 6.9), were incubated with heme (0.2 to 40 µM) in the dark at room temperature for 60 minutes before UV/Vis spectra were measured at 300 to 600 nm. Protein concentrations were corrected using the calculated molar absorption coefficient at 280 nm. Peptides **1–7** (20 µM) were incubated with heme for 30 minutes in the dark at room temperature^9^.

### Fluorescence spectroscopy

All measurements were performed on a fluorescence spectrophotometer FP-8300 (Jasco, Tokyo, Japan). BSA (1 µM) and lysozyme (1 µM) were dissolved in PBS buffer (pH 7.4) and IL-36α variants (1 µM) in 20 mM sodium phosphate buffer (pH 6.9), were each dissolved in and incubated for 60 minutes with varying heme concentrations (0.1–16 µM) in the dark. Parameters were set to excitation at 306 nm, emission at 352 nm, bandwidth 10 nm (extinction) and 20 nm (emission)^30^.

### Resonance Raman spectroscopy

Resonance Raman spectroscopic measurements were conducted utilizing a Horiba Jobin-Yvon LabRam HR 800 Raman spectrometer (Horiba, Kyoto, Japan) equipped with a back-illuminated deep-depletion CCD detector (1024 × 256 pixels) cooled by liquid nitrogen. For excitation of Raman scattering, a Coherent Innova 300 C ion laser using the krypton line at 413.1 nm was applied. The Raman system was connected to an Olympus BX41 upright microscope (Olympus, Tokyo, Japan) with a motorized XY microscope stage and a 20x objective (Olympus UPlanFL N, NA 0.50) which served to focus the incoming laser light onto the sample as well as for the collection of the 180° backscattered light. Aqueous solutions (phosphate buffered NaCl, pH 7.0) of heme (400 µM) and peptide or protein were equimolarly mixed. The heme solution used here was prepared using a protocol established in earlier studies^9^. The reaction mixture was incubated for 30 min (for peptides) or 60 min (for proteins) in the dark at room temperature and centrifuged to remove precipitant before measurement of the supernatant solution.

### Circular dichroism (CD) spectroscopy

The secondary structure content of IL-36α was estimated using the algorithms CAPITO (CD Analysis and Plotting tool)^52^ and K2D3^53^. CAPITO takes advantage of the available validated protein CD spectra in the Protein Circular Dichroism Data Bank (PCDDB) repository, while the K2D3 analysis is based on the k-nearest neighbors algorithm for pattern recognition. The wavelength range used for K2D3 was 190–240 nm. The data obtained from the programs mentioned is shown in Supplementary Table S6.

### NMR structure analysis

Solution NMR experiments were performed at 283 K on Bruker Avance III spectrometers with proton frequencies of 750 and 600 MHz. The IL-36α samples were dissolved in 20 mM phosphate buffer with 65 mM NaCl and 5% D_2_O using the freeze-dried solid compound. For NMR studies of the heme-bound form, Ga(III)-protoporphyrin IX chloride (data not shown) and heme were used as obtained from Frontier Scientific (Logan, USA). Protein [^1^H,^15^N]-HSQC spectra in the free and heme-bound state were recorded at concentrations of 100 µM for trIL-36α and of 140 µM for wild type IL-36α. NMR data were acquired and processed with Topspin (Bruker, Rheinstetten, Germany) and analyzed with XEASY^54^. Based on heteronuclear 3D NMR spectroscopy, an almost complete resonance assignment could be achieved^29^. Distance constraints were extracted from a NOESY spectra acquired with 120 ms mixing time. Upper limit distance constraints were calibrated according to their intensity in the NOESY spectrum. Cross peaks of vicinal and geminal protons were used as calibration reference. Torsion angle constraints were obtained from local conformational analysis with the FOUND module^55^. In addition, torsion angle constraints defining the allowed ϕ,ψ-regions in the Ramachandran map were included. The 20% of structures with the lowest CYANA target functions^56^ were selected to represent the NMR solution structures. The figures were produced using MOLMOL^57^.

### Creation of IL-36α mutant structures

The structures of the seven IL-36α proteins mutants were created based on the NMR solution structure of IL-36α using the *Mutator* plugin of the VMD^58^ program (version 1.9.3). Point mutations were iteratively introduced to the starting IL-36α NMR structure replacing Cys and Tyr residues by Ser and His and Pro residues by Ala. A single residue was mutated at each iteration and the resultant mutated structure was energy minimized by a simulated annealing energy minimization protocol using the Yasara (version 18.2.7)^59^ molecular modeling and simulation suite. In this manner, the seven mutants of IL-36α namely Y108S, H109A, Y108SH109A, C136SP137A, Y108SC136SP137A, H109AC136SP137A, and Y108SH109AC136SP137A were generated. All final mutant structures were energy minimized before being used for molecular docking and molecular dynamics (MD) simulations.

### Molecular dynamics simulations

MD simulations were run using the MD macro in Yasara^60^ (Yasara structure version 18.2.7)^30^. All simulations were run with a 2 fs time step using the AMBER99SB-ILDN force field^61^. Force field parameterization of the simulated system was done by the AutoSMILES method (http://www.yasara.org/autosmiles) implemented in Yasara. This entails parameter assignment for the protoporphyrin system using GAFF^62^ and AM1-BCC^63^ with Fe(III) vdW parameters taken from Li *et al*.^64^ as reported previously^30^. The protein (or the heme-protein complex) was placed in the center of a cubic simulation cell with a distance of at least 15 Å from the edge of the box. The cell was filled with the 3 point model^65^ of water with a physiological concentration of 0.9% NaCl. Periodic boundary conditions were used and a cut-off for long range was set at 8 Å. Long range coulomb interactions were accounted for by the particle-mesh Ewald^66^ method. All simulations were run in the NPT ensemble at 298 K and the pressure maintained at 1 atm for 200 ns. Molecular graphics were produced using VMD and plots using Grace version 5.1.25 (http://plasma-gate.weizmann.ac.il/Grace/).

### Molecular docking simulations

Molecular docking simulations were conducted using Yasara software^59^ and the ensemble docking method^67^ implemented in the program. A receptor ensemble of 20 high scoring side chain conformations at 298 K was created by the program on which the ligand heme (ChemSpider^68^, 16739951) was docked 400 times. This resulted in 8,000 runs per docking experiment. The search space for docking H109 was narrowed to a 10 Å radius around H109, whereas the full structure of the protein was covered as the docking search space for all other runs. Results from docking runs were scored by predicted binding energies. A clustering method that employs a 5 Å heavy atom RMSD threshold between docked conformations was used to sort the final set of docked complexes. The top five complexes ranked by predicted binding energies were subject to closer investigation.

### Isolation of human fibroblast-like synoviocytes

Human fibroblast-like synoviocytes (FLS) were isolated from knee joints of rheumatoid arthritis patients obtained at the University Hospital Erlangen-Nuremberg^69^. All patients gave written informed consent, and their use for research was approved by the ethics committee of the University hospital Erlangen (ethic licenses 4013 and 4065). We confirm that all methods were performed in accordance with the relevant guidelines and regulations. Human RA FLS kept at 37 °C and 5% CO_2_ in a humidified incubator upon isolation. They were cultured in RPMI and DMEM at a ratio of 1:5, supplemented with 50 U/mL penicillin, 50 μg/mL streptomycin, 0.2% amphotericin and 2% fetal bovine serum. Cells between passages 3 to 8 were used.

### Stimulation of RA FLS with trIL-36α, mutants and heme

For stimulation experiments, human FLS from RA patients were seeded at a concentration of 100,000 cells/mL in 500 µL culture medium in a 48-well plate one day prior to the experiment. Before stimulation of FLS, heme was pre-incubated with trIL-36α or trIL-36α protein mutants with substituted amino acids in the proposed heme-binding site in medium for 1 h at room temperature in the dark. Herein, the concentration of trIL-36α or trIL-36α mutants was kept constant at 5.8 nM with varying ratios of IL-36α to heme (1:26 and 1:260, i.e. 0.15 µM and 1.5 µM). Cells were then incubated for either 5 min (western blot analysis of phosphorylation) or 24 h (qPCR and ELISA). Sample separation was performed by a reducing 2D gel electrophoresis (10% SDS polyacrylamide gel) and blotted to a nitrocellulose membrane by semi-dry transfer. Blocking was performed using 5% BSA/TBST for 1 h at room temperature, before the blots were incubated overnight at 4 °C with antibodies (rabbit anti-P-p38, rabbit anti-p38 and rabbit anti-GAPDH) from Cell Signaling Technology (Leiden, the Netherlands). As secondary antibody anti-rabbit HRP (Biozol, Eching, Germany) was applied. A Pierce™ ECL Western Blotting Substrate (Thermo Fisher Scientific, Dreieich, Germany) was used for detection according to the manufacturer’s instruction. Western Blot images were acquired in the chemiluminescence-imager CELVIN® S (Biostep, Burkhardtsdorf, Germany) with the software SnapAndGo (Version 1.6.1). Protein detection parameter are: p38 (Exposure time 10 min, dynamic range 1 to 12,080), P-p38 (Exposure time 110 min, dynamic range 1 to 12,080) and GAPDH (Exposure time 6 min, dynamic range 1–12,080). Cell viability was analyzed using the AlamarBlue assay according to the manufacturer’s instruction (#741802, Invitrogen, Carlsbad, CA, USA), and absorbance was measured at 570 nm and 600 nm.

### RNA, cDNA preparation and quantitative RT-PCR

For performing quantitative real-time PCR (qRT-PCR), RNA was isolated from FLS with peqGold TriFast (Peqlab, Erlangen, Germany), followed by first-strand cDNA synthesis using the MultiScribe™ MuLV reverse transcriptase (Applied Biosystems, Foster City, CA, USA), both according to manufacturer’s instruction. Relative gene expression was assessed by qRT-PCR using the Applied Biosystems 7500 fast-real-time-PCR System (Applied Biosystems) with SYBR® Select Master Mix (ThermoFisher Scientific) as detection method, according to the manufacturer’s manual. Samples and the housekeeping gene GAPDH as endogenous control were analyzed in duplicates. For evaluation, relative expression (ΔCt) and ΔΔCt method was utilized. Primers used were GAPDH (fwd 5′-TCCTGTTCGACAGTCAGCCGC-3′, rev 5′-CGCCCAATACGACCAAATCCGT-3′), IL-6 (fwd 5′-AGAGCTGTGCAGATGAGTACAA-3′, rev 5′-GCGCAGAATGAGATGAGTTGTC-3′) and IL-8 (fwd 5′-AGCACCAGCCAACTCTCACT-3′, rev 5′-CGTTAACTGCATCTGGCTGA-3′).

### Enzyme-linked immunosorbent assay (ELISA)

ELISAs were performed with DuoSet ELISA Kits (R&D Systems, Minneapolis, MN, USA) according to manufacturer’s instructions, for which FLS cell culture supernatants were used undiluted. The optical density at 450 nm, with a wavelength correction set to 540 nm, was evaluated using the SpectraMax 190 ELISA-Reader and the Software Softmax Pro Version 3.0 (both Molecular Devices, Sunnyvale, CA, USA).

### Bioinformatics

Values for sequence identity and similarity/homology were calculated with the SIAS webserver^[SECRETARIA GENERAL DE CIENCIA, TECNOLOGIA E INNOVACION OF SPAIN]^. Protein sequences for analysis and alignment were retrieved from Uniprot^70^. Alignments were carried out using the Clustal Omega webserver^31^.

## Quantitation and Statstical Analysis

Statistical readout was performed using Graph Pad Prism 4.00 software (La Jolla, USA). Group differences were considered statistically significant with a p-value less than 0.05. Statistical parameters are reported in the figure legends.

## Supplementary information

Supplementary Information

Video 1

Video 2

## Data Availability

Atomic coordinates of IL-36α have been deposited in the Protein Data Bank (http://www.wwpdb.org/) under PDB: 6HPI.
