# Supplementary material for: Structural insights into heme binding to IL-36α proinflammatory cytokine
Source: Sci Rep. 2019 Nov 15;9:16893. doi: 10.1038/s41598-019-53231-0 (PMC6858345; doi:10.1038/s41598-019-53231-0)
Supplement: Supplementary file 1 — Supplementary Information [file 41598_2019_53231_MOESM1_ESM.pdf]

Supplementary Information

Structural insights into heme binding to IL-36α proinflammatory cytokine

Amelie Wißbrock[a,1], Nishit B. Goradia[b,1,2], Amit Kumar[b], Ajay Abisheck Paul George[a], Toni Kühl[a], Peter Bellstedt[c], Ramadurai Ramachandran[b], Patrick Hoffmann[d,e], Kerstin Galler[d,e], Jürgen Popp[e,f], Ute Neugebauer[d,e,f], Kornelia Hampel[g], Bastian Zimmermann[g], Susanne Adam[h], Maximilian Wiendl[h], Gerhard Krönke[h], Iqbal Hamza[i,j], Stefan H. Heinemann[k], Silke Frey[h], Axel J. Hueber[h], Oliver Ohlenschläger[b,*], and Diana Imhof[a,*,]

[a]Pharmaceutical Biochemistry and Bioanalytics, Pharmaceutical Institute, University of Bonn, D-53121 Bonn, Germany;

[b]CS Protein Production, Leibniz Institute on Aging/Fritz Lipmann Institute, D-07745 Jena, Germany;

[c]Institute of Organic and Macromolecular Chemistry (IOMC), Friedrich Schiller University Jena, D-07743 Jena, Germany;

[d]Center for Sepsis Control and Care (CSCC), Jena University Hospital, D-07747 Jena, Germany;

[e]Leibniz Institute of Photonic Technology (Leibniz IPHT), D-07745 Jena, Germany;

[f]Institute of Physical Chemistry and Abbe Center of Photonics, Friedrich Schiller University Jena, D-07743 Jena, Germany;

[g]Biaffin GmbH & Co KG, D-34132 Kassel, Germany;

[h]Department of Internal Medicine 3 – Rheumatology and Immunology, University of Erlangen-Nürnberg (FAU) and University Hospital Erlangen, D-91054 Erlangen, Germany;

[i]Department of Animal & Avian Sciences, University of Maryland, College Park, MD 20742;

[j]Department of Cell Biology & Molecular Genetics, University of Maryland, College Park, MD 20742;

[k]Center for Molecular Biomedicine, Department of Biophysics, Friedrich Schiller University Jena and Jena University Hospital, D-07745 Jena, Germany;

[1]These authors contributed equally.

[2]Current address: European Molecular Biology Laboratory, D-22607 Hamburg, Germany.

*Correspondence: dimhof@uni-bonn.de, oliver.ohlenschlaeger@leibniz-fli.de

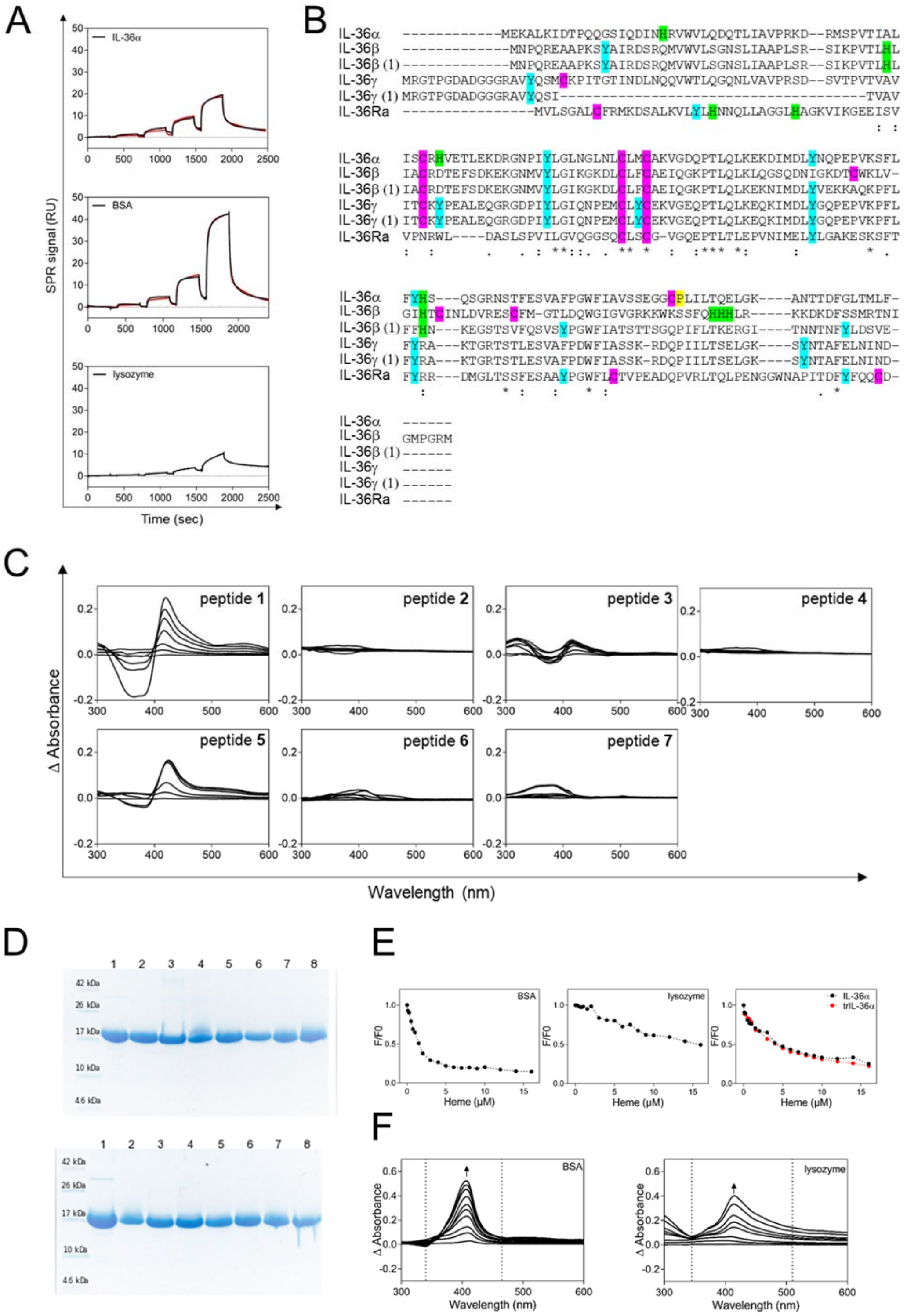
A
IL-36α
BSA
lysozyme
SPR signal (RU)
Time (sec)
B
IL-36α ------------MEKALKIDTPQQGSIQDINHRVWVLQDQTLIAVPRKD--RMSPVTIAL
IL-36β -------------MNPQREAAPKSYAIRDSRQMVWVLSGNSLIAAPLSR--SIKPVTLHL
IL-36β (1) -------------MNPQREAAPKSYAIRDSRQMVWVLSGNSLIAAPLSR--SIKPVTLHL
IL-36γ MRGTPGDADGGGRAVYQSMCKPITGTINDLNQQVWTLQGQNLVAVPRSD--SVTPVTVAV
IL-36γ (1) MRGTPGDADGGGRAVYQSI-------------------------------------TVAV
IL-36Ra ----------------MVLSGALCFRMKDSALKVLYLHNNQLLAGGLHAGKVIKGEEISV
IL-36α ISCRHVETLEKDRGNPIYLGLNGLNLCLMCAKVGDQPTLQLKEKDIMDLYNQPEPVKSFL
IL-36β IACRDTEFSDKEKGNMVYLGIKGKDLCLFCAEIQGKPTLQLKLQGSQDNIGKDTCWKLV-
IL-36β (1) IACRDTEFSDKEKGNMVYLGIKGKDLCLFCAEIQGKPTLQLKEKNIMDLYVEKKAQKPFL
IL-36γ ITCKYPEALEQGRGDPIYLGIQNPEMCLYCEKVGEQPTLQLKEQKIMDLYGQPEPVKPFL
IL-36γ (1) ITCKYPEALEQGRGDPIYLGIQNPEMCLYCEKVGEQPTLQLKEQKIMDLYGQPEPVKPFL
IL-36Ra VPNRWL----DASLSPVILGVQGGSQCLSCG-VGQEPTLTLEPVNIMELYLGAKESKSFT
IL-36α FYHS---QSGRNSTFESVAFPGWFIAVSSEGGCPLILTQELGK----ANTTDFGLTMLF-
IL-36β GIHTCINLDVRESCFM-GTLDQWGIGVGRKKWKSSFQHHHLR-----KKDKDFSSMRTNI
IL-36β (1) FFHN---KEGSTSVFQSVSYPGWFIATSTTSGQPIFLTKERGI----TNNTNFYLDSVE-
IL-36γ FYRA---KTGRTSTLESVAFPDWFIASSK-RDQPIILTSELGK----SYNTAFELNIND-
IL-36γ (1) FYRA---KTGRTSTLESVAFPDWFIASSK-RDQPIILTSELGK----SYNTAFELNIND-
IL-36Ra FYRR---DMGLTSSFESAAYPGWFLCTVPEADQPVRLTQLPENGGWNAPITDFYFQQCD-
IL-36α ------
IL-36β GMPGRM
IL-36β (1) ------
IL-36γ ------
IL-36γ (1) ------
IL-36Ra ------
C
peptide 1
peptide 2
peptide 3
peptide 4
peptide 5
peptide 6
peptide 7
Δ Absorbance
Wavelength (nm)
D
42 kDa
26 kDa
17 kDa
10 kDa
4.6 kDa
E
BSA
lysozyme
IL-36α
trIL-36α
F/F0
Heme (μM)
F
BSA
lysozyme
Δ Absorbance
Wavelength (nm)

Figure S1. Spectroscopic studies on heme-IL-36α and heme-peptide complexes. (A) Surface plasmon resonance (SPR) spectra of heme-incubated IL-36α and controls (BSA, lysozyme; spectra in black, data fits in red). A K_D of 6 to 11 μM for the first and 30 to 41 μM for the second binding event was determined for BSA. It was not possible to determine the K_D of lysozyme due to weak signals. Lysozyme was thus considered as non-binder as earlier described (Shen et al., 2014). **(B)** Sequence alignment of IL-36α, β, γ (including all isoforms), and IL-36Ra by Clustal Omega (Sievers et al., 2011)**.** Potential heme-coordinating residues are marked in blue (Y), green (H), and purple (C). IL-36α is the only variant with a CP motif (P137 in yellow), whereas other amino acids were identified in IL-36β (isoform 1: K136S137, isoform 2: Q135P136) and IL-36γ (isoform 1: Q147P148, isoform 2: Q112P113) at the same position. From the ten potential HRMs in IL-36α, several motifs can be excluded due to their location and/or negative net charge as was earlier defined (Supplementary Table S2)[10,11].

(C) UV/Vis differential spectra for heme-incubated IL-36α-derived peptides **1-7**. Peptides **2** and **4** (Cys-mutants) as well as **6** (Y108AH) and **7** (Y108AH109A) displayed no heme binding thereby verifying the essential role of Cys (**2**, **4)** and Tyr (**6**, **7**). Peptide **3** (Cys only) exhibited a maximum shift to ~319 nm and to ~416 nm (K_D determination was not possible). Heme binding to peptide **1** (YH) revealed a UV/Vis band shift to ~415 nm with a K_D value of 4.48 ± 2.20 μM. Peptide **5** (YH109A) showed a UV/Vis band shift to ~418 nm with a K_D value of 2.29 ± 1.32 μM. A different binding mode of peptides **1** and **5** is visible from the diverging curve shape and a loss of affinity (**5**) confirms an impact of Tyr and partially His in wild-type peptide **1**. **(D)** SDS PAGE of IL-36α proteins (Coomassie blue-stained 18% reducing gel). The proteins (left: full-length, right: truncated) are ordered as follows: 1) wild-type, 2) Y108S, 3) H109A, 4) C136SP137A, 5) Y108SH109A, 6) Y108SC136SP137A, 7) H109AC136SP137A, and 8) Y108SH109AC136SP137A. Molecular weight markers are indicated. **(E)** Fluorescence intensity of heme-incubated controls (BSA, lysozyme) as well as IL-36α and trIL-36α (λ_{ex} 306 nm, λ_{em} 352 nm). Heme-protein complex formation resulted in static quenching (hyperbolic decay function) in contrast to dynamic quenching (linear decay function) as previously described (Peherstorfer et al., 2018). Lysozyme was confirmed as non-binder (static quenching only) and BSA as binder (dynamic and static quenching) as

reported earlier (Shen et al., 2014). For IL-36α a similar trend as found for BSA was observed. **(F)** Differential absorption spectra of heme-incubated (0-40 μM) BSA and lysozyme. A broad band as found for lysozyme hints at unspecific heme-protein interaction (Peherstorfer et al., 2018).

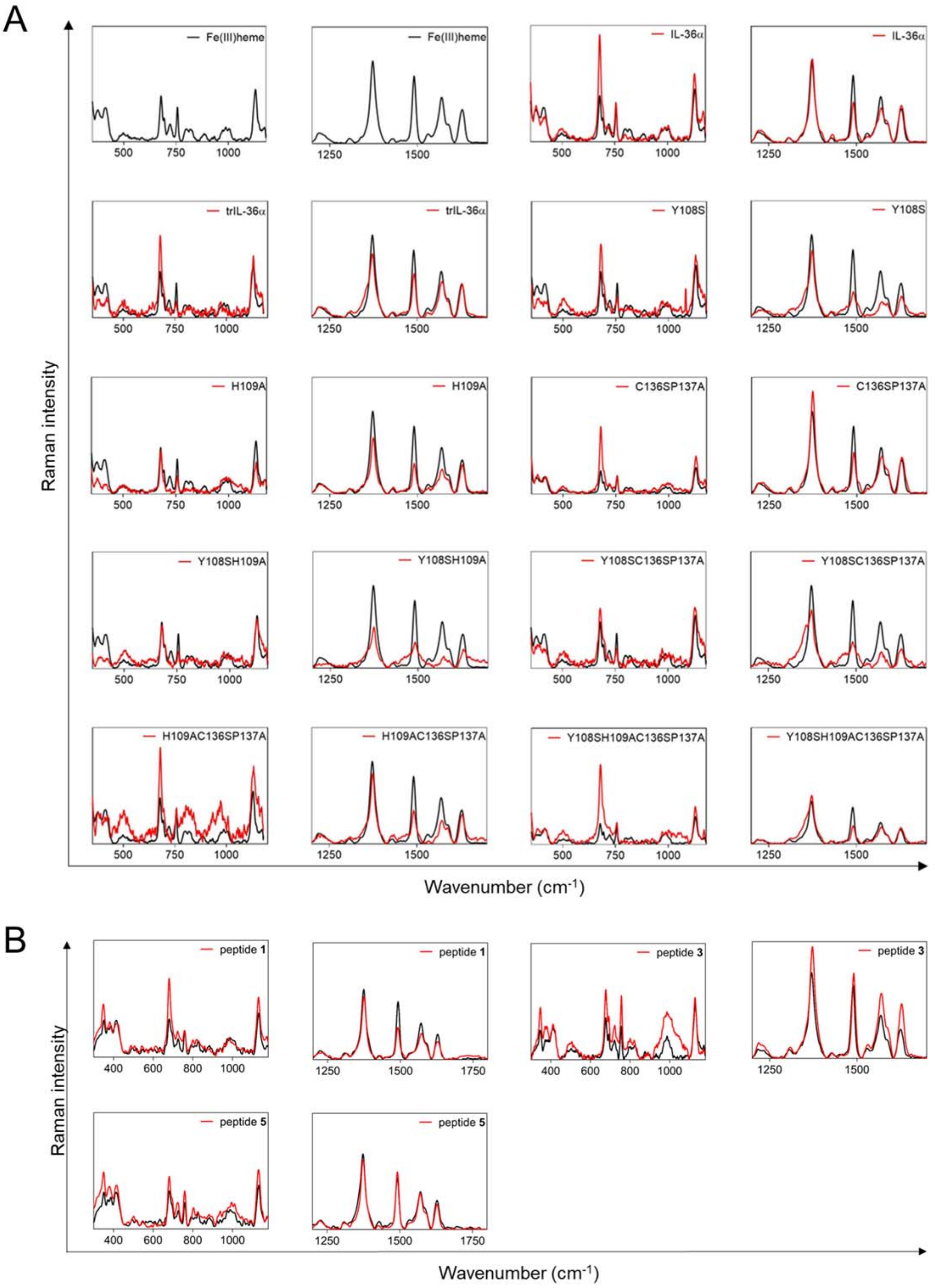

A
Fe(III)heme
IL-36α
trIL-36α
Y108S
H109A
C136SP137A
Y108SH109A
Y108SC136SP137A
H109AC136SP137A
Y108SH109AC136SP137A
Raman intensity
Wavenumber (cm-1)
B
peptide 1
peptide 3
peptide 5
Raman intensity
Wavenumber (cm-1)

Figure S2. Resonance Raman spectrosopcy of IL-36α, mutant proteins, and IL-36α-derived peptides. (A) Resonance Raman spectra of heme (in black), wild-type (tr)IL-36α, and IL-36α protein mutants (all proteins in red). Lower (left) and higher (right) wavenumber fingerprint region with assignment of prominent normal mode frequencies ν_7 (681 cm^{-1}), ν_4 (1374 cm^{-1}), ν_3 (1492 cm^{-1}), ν_2 (1571 cm^{-1}), and ν_{10} (1628 cm^{-1}) for heme. To increase the visibility of the spectra at low wavenumbers, lower wavenumbers an adapted y-axis scaling was applied. For the C136SP137A mutant a decrease in intensity of the ν_3 and ν_2 bands and an increase of intensity of the vibrational mode ν_7 (around 681 cm^{-1}) as well as the oxidation state marker band ν_4 (around 1374 cm^{-1} for Fe(III)) might indicate the negligible formation of a hexacoordinated heme-complex (Supplementary Table S1)[27]. Yet, this behavior was neither present in all four mutants of the CP motif nor occurred it to the same extent and thus should be interpreted with caution. **B)** Resonance Raman spectra of heme-incubated peptides **1**, **3,** and **5**. Data anlaysis was performed as described in (A). Peptide **3** was measured in another experiment than the other complexes.

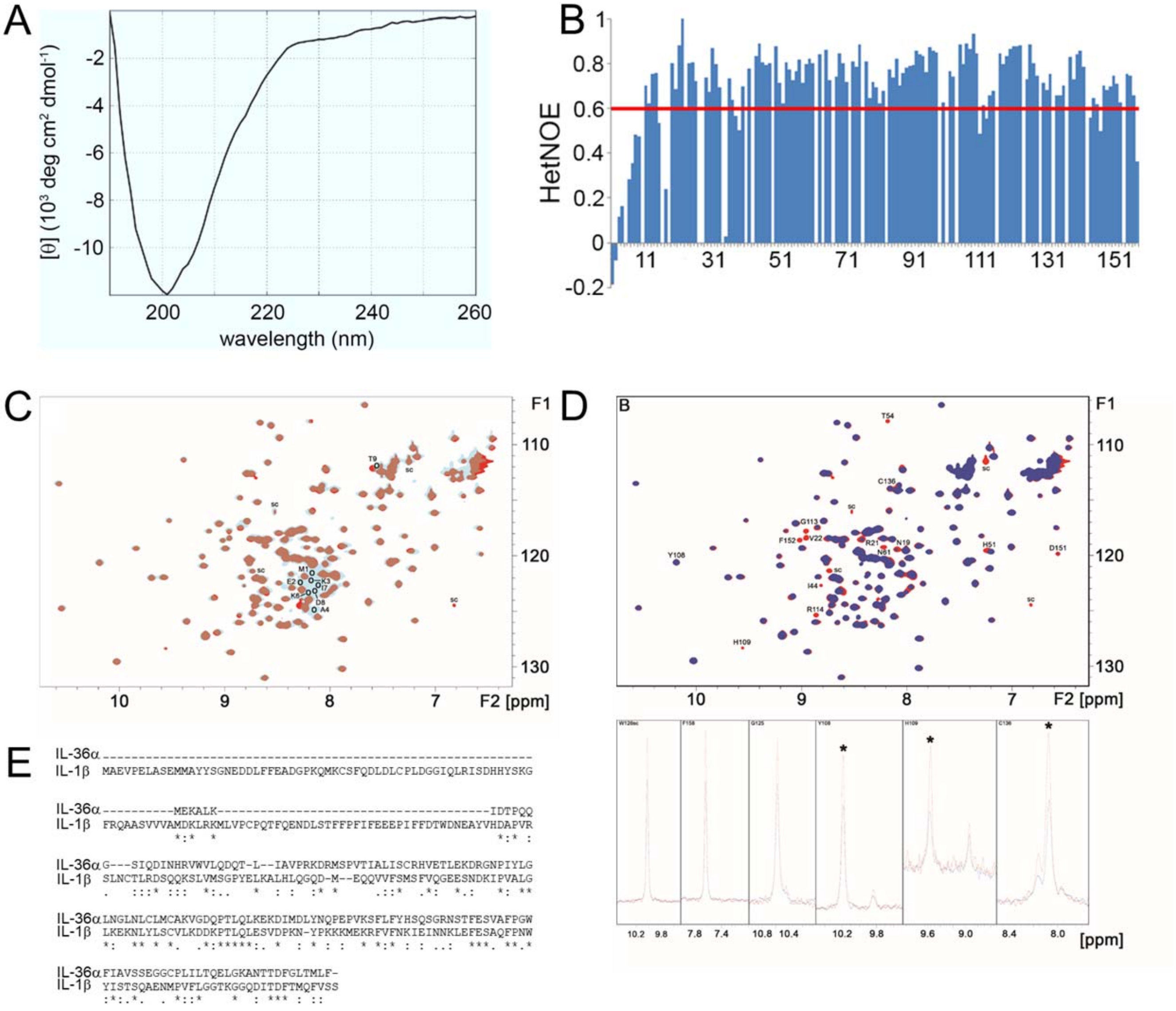

Figure S3. Structural analysis of IL-36α in free and heme-bound form. (A) The negative band at 203 nm in the CD spectrum of IL-36α suggests a rather disordered secondary structure (Table S6). The prediction for wild-type IL-36α can be summarized as a minor content of α-helix (~ 10%), between 20 and 35% β-sheet fold, and a large fraction (~ 50-70%) of irregular structural elements. **(B)** The {^{1}H,^{15}N} heteronuclear NOE data of IL-36α provides information about the motion of the N-H bond vectors and thus the protein backbone dynamics. Values below 0.6 indicate an increased mobility. **(C)** Comparison of the [^{1}H,^{15}N]-HSQC spectra of full-length (cyan) and truncated IL-36α (red). Missing or

shifted signals of the N-terminal residues in trIL-36α are indicated by black circles (sc ~ side chain signal). **(D)** Structural analysis of heme binding to trIL-36α. Top: Superposition of the [^{1}H,^{15}N]-HSQC spectra of trIL-36α in the free (red) and heme-bound state (blue). A drastic decrease in signal intensities is observed for residues N19, R21, V22, I44, H51, T54, N61, H109, G113, R114, D151, and F152 (sc ~ side chain signal). Bottom: Moderate changes compared to e.g. H109 apply to Y108 and C136 (relevant cross peaks marked by *). For comparison of the uniform drop in signal intensities as a result of the solvent paramagnetic effect, the traces of the binding-independent residues F158 and G125 as well as the side chain signal of W126 are given on the left. **(E)** Sequence alignment of IL-36α and IL-1β was performed using Clustal Omega (Sievers et al., 2011).

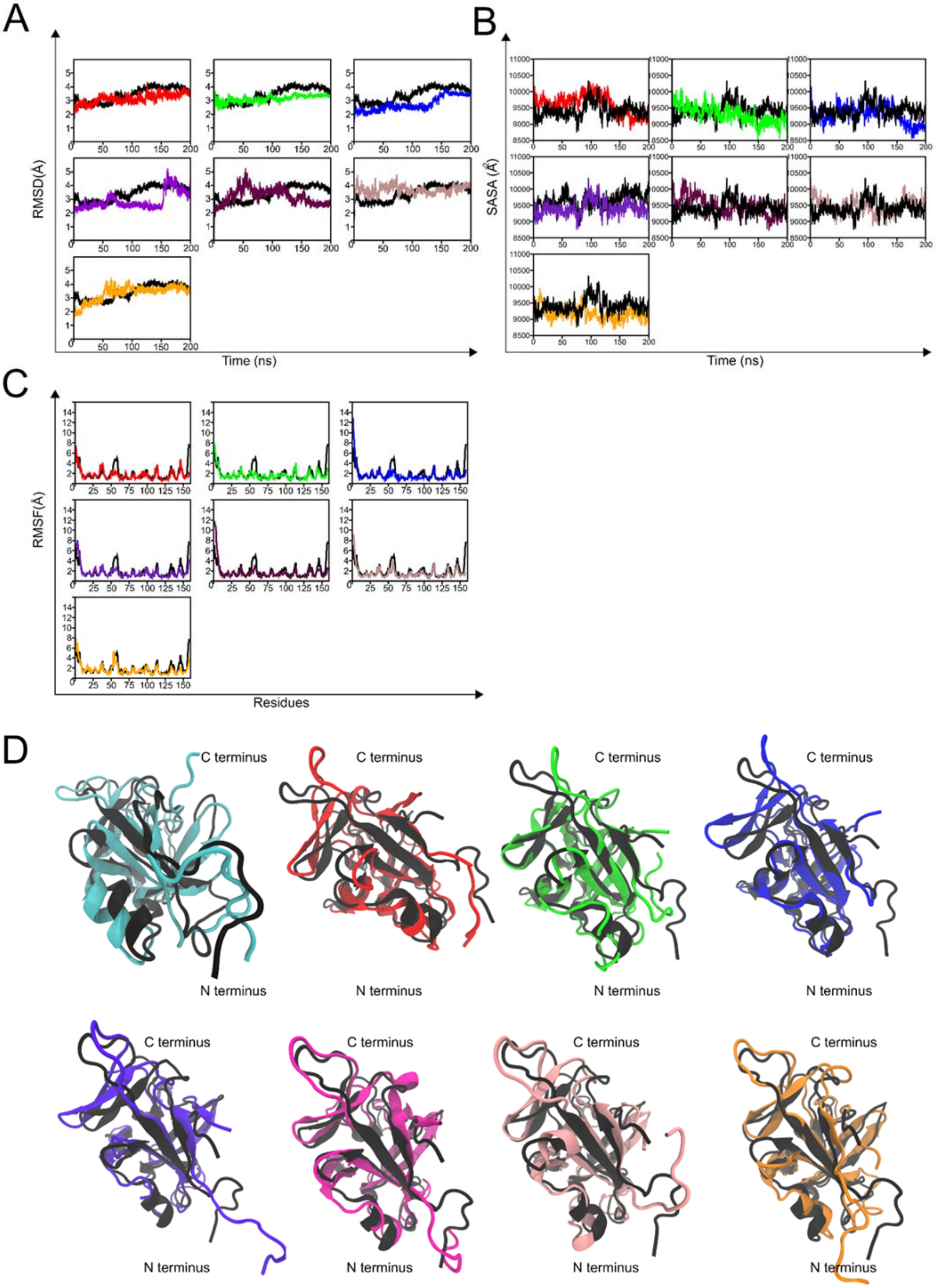

A
RMSD(Å)
Time (ns)
B
SASA (Å)
Time (ns)
C
RMSF(Å)
Residues
D
C terminus
N terminus

Figure S4. Computational studies on IL-36α wildtype and mutant proteins. Protein mutants in A-C are presented as follows: Y108S (red), H109A (green), Y108SH109A (blue), C136SP137A (indigo), Y108SC136SP137A (maroon), H109AC136SP137A mutant (brown), and Y108SH109AC136SP137A (orange). **(A)** Backbone RMSD (root mean square deviation) generated from the MD simulations. The IL-36α proteins reach sufficient equilibration at around 150 ns of simulation time. The average RMSD change of the wild-type IL-36α structure with reference to the NMR starting structure was 3.34 Å (Table S7). This structural deviation largely arises from the extreme fluctuations exhibited by the residues at the C- and N-terminal regions of the protein, as can be seen from the RMSF (root mean square fluctuation per residue) plots in **(B)**. The residues at the termini fluctuate at about 8 Å from their mean positions which has a consequent impact on the overall RMSD value. The conformational ensemble produced by the MD simulations are a suitable representation of the NMR ensemble of the protein given that the NMR structures differ from each other by RMSD values up to 2.74 Å (data not shown). The disordered regions of the N-terminal region in the NMR ensemble are also seen in the conformations sampled in MD simulations and are quantified by the high RMSF values associated with them. **(C)** SASA (solvent accessible surface area) comparison between wild-type and mutant IL-36α structures show evolution of the SASA to lower values for the Y108SH109A mutant and higher values for the C136SP137A mutants and no significant deviation from the wild-type in the other mutants. This is a logical consequence of replacing residues with higher mean SASA values (46 Å2 for Y and 54 Å2 for H) by ones with lower values (39 Å2 for S and 28 Å2 for A). The increase of SASA for the C136P137A mutants can also be explained similarily given that the mean SASA values for C (17 Å2) is much lower than that of S (39 Å2). The SASA values (Y-axis) start from 8500 onwards to avoid visual obfuscation. **(D)** Structural superimposition over Cα atoms of the NMR starting structure of IL-36α (black) and the final frame from the MD simulations of the IL-36α wild-type (cyan, A) and mutants Y108S (red), H109A (green), Y108SH109A (blue), C136SP137A (violet), Y108SC136SP137A (magenta), H109AC136SP137A (pink), and Y108SH109AC136SP137A (orange). All structures are represented by the "new cartoon" style in VMD (Visual Molecular Dynamics) (Humphrey et al., 1996). MD simulations of the heme-bound

complexes revealed that C136-mediated heme binding was a prerequisite for stable coordination of heme via Y108. As observed from the RMSF profiles, heme binding to C136 supported by P137 and other surrounding residues reduced the conformational flexibility of loops 6, 10, and some parts of loop 3 (Figures 4B, D). This binding-induced constraint has a dampening effect on the flexibility of the rest of the protein rendering it conformationally favorable for the binding event via Y108. Although heme coordination via Y108 is still observed upon loss of the CP motif, MD simulations of the complex resulted in heme dislocation from the Y108 binding site into solution in about 5 ns of the simulation (***Movie S1***). With heme bound to C136 (e.g. wild-type IL-36α), however, the binding of the second heme molecule to Y108 persists for a much longer period of time (~150 ns in 200 ns MD simulations, ***Movie S2***). To investigate coordination via the H109 residue, heme was docked to the protein by narrowing down the docking search space to the vicinity of H109. Yet, MD simulations revealed almost instantaneous dislocation of heme from the protein surface suggesting a lower preference of heme to bind via H109 over Y108. Apart from heme binding via the proposed motifs, an additional heme interaction was observed for all proteins investigated. This interplay was characterized by the Fe(III) ion found at a distance 2.42 Å from the oxygen atom of the S104 residue. This observation might explain heme binding to the Y108SH109AC136SP137A mutant as found by UV/Vis spectroscopy. This S104-mediated attachment of heme may have an influence on the protein by inducing a restraining effect on the protein's conformational flexibility. Although no coordinative interactions were observed, the electrostatic and hydrophobic interactions between heme and the protein surface at this interface may facilitate a surface attachment.

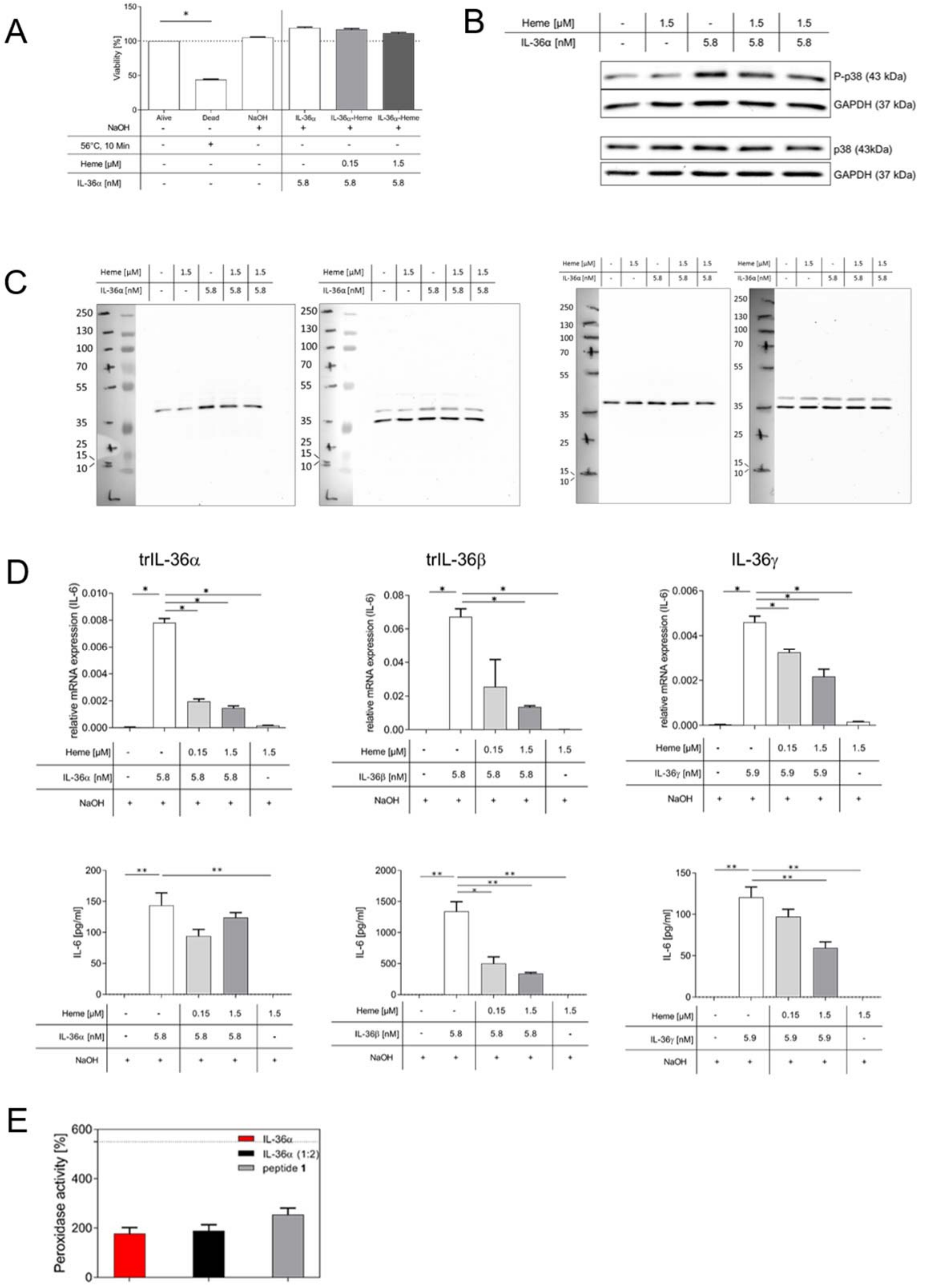
A
Viability [%]
Alive
Dead
NaOH
IL-36α
IL-36α-Heme
56°C, 10 Min
Heme [μM]
IL-36α [nM]
B
Heme [μM]
IL-36α [nM]
P-p38 (43 kDa)
GAPDH (37 kDa)
p38 (43kDa)
C
D
trIL-36α
trIL-36β
IL-36γ
relative mRNA expression (IL-6)
IL-6 [pg/ml]
IL-36β [nM]
IL-36γ [nM]
E
Peroxidase activity [%]
IL-36α (1:2)
peptide 1

Figure S5. Biological testing of the agonistic trIL-36α family members. (A) Cell viability upon treatment of synovial fibroblast-like synoviocytes (FLS) from RA patients with trIL-36α, heme, and the trIL-36α-heme complex for 24 h. IL-36α and the respective agent were preincubated for 1 h prior to the addition to FLS. Cell viability was analyzed by the AlamarBlue assay according to the manufacturer's instruction (#741802, Invitrogen, Carlsbad, CA, USA). Statistical analysis of samples with reduced cell viability compared to the alive control was performed using Wilcoxon matched-pairs signed rank test with $*p < 0.05$. Values are means + SEM (n=6). (B) Impact of heme on IL-36α-induced intracellular signaling in FLS from RA patients. FLS were treated with varying trIL-36α to heme ratios for 5 min. trIL-36α was preincubated with heme for 1 h prior to addition to the FLS. Western blot analysis was performed using antibodies such as rabbit anti-P-p38, rabbit anti-p38, and rabbit anti-GAPDH (Cell Signaling Technology, Leiden, the Netherlands). As secondary antibody anti-rabbit HRP (Biozol, Eching, Germany) was used. Detection was performed using Pierce™ ECL Western Blotting Substrate (Thermo Fisher Scientific, Dreieich, Germany) according to the manufacturer's instruction. Shown is one representative (of n=4) Western blot for phosphorylated p38 (P-p38) and p38 with respective GAPDH loading control. Western Blots were cropped for clarity and full-length blots are presented in (C). (D) Heme reduces trIL-36α, β, and γ-induced gene expression in FLS from RA patients. FLS were treated with varying ratios of recombinant human IL-36α, IL-36β, and IL-36γ (R&D Systems, Minneapolis, MN) to heme for 24 h. The left panel shows the relative mRNA expression of IL-6 normalized to GAPDH as analyzed by qRT-PCR, whereas the right panel illustrates the concentration of IL-6 in the cell culture supernatant as measured by ELISA. Values are means + SEM (n=5). Statistical analysis was performed using Mann-Whitney test with $*p < 0.05$ and $**p < 0.01$. (E) Peroxidase-like activity (Atamna and Boyle, 2006; Atamna and Frey, 2004; Ghosh et al., 2015; Wißbrock et al., 2017) of heme-incubated IL-36α (1:1 and 1:2, protein:heme) and peptide 1. No significant increase of the heme's peroxidase activity (100%) was observed which is in agreement with the results obtained for the CP-based HRM on peptide level in previous studies (Wißbrock et al., 2017).

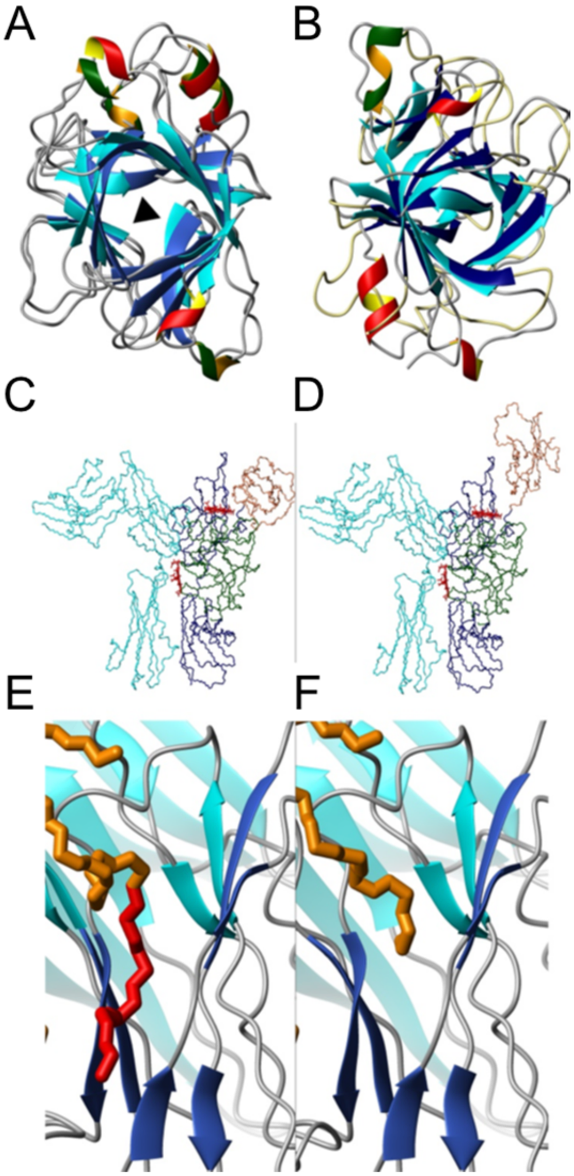

Figure S6. IL-36α structural comparison to IL-36γ and IL-1β as well as IL-36α superimposition with the IL-1β receptor. (A) Superimposition of the NMR structure of IL-36α and IL-36γ (PDB code: 4IZE). The NMR structure of IL-36α (β-strands cyan; helices red/yellow) and the X-ray structure of IL-36γ (β-strands navy; helices green/orange) are displayed. The trefoil pseudo-D3 symmetry axis is indicated by a black triangle. **(B)** Superimposition of the NMR structure of IL-36α and IL-1β (PDB code: 3O4O). The NMR-structure of IL-36α (β-strands cyan; helices red/yellow, backbone grey) and the X-ray structure of IL-1β (β-strands navy; helices green/orange; backbone khaki) are displayed.

(C) Superimposition of the NMR solution structure of IL-36α with the IL-1β in the receptor complex (PDB code: 3O4O). **(C, D)** View of the full complex model. The wild-type N-terminal residues and loop 3 (red) show steric clashes with the IL-1RII domain-III and domain-I, respectively. **(E, F)** Truncation of the IL-36α sequence by the N-terminal five amino acids allows to resolve the steric interactions with two β-strands of IL-1RII (E) and to accommodate the flexible N-terminal stretch into a V-shaped cleft of IL-1RII domain-III (F). Rotation about the torsion angle ϕ of Asp315 in IL-1RII leads to a repositioning of the full IL-1RII domain-I thereby resolving van der Waals clashes with loop 3 of IL-36α. Coloring: IL-36α – green, IL-1RII domain-I – coral, IL-1RII domain-II and domain-III – navy, IL-1RAcP – cyan, IL-36α cysteine-136 – yellow, IL-36α loop 3, and N-terminal residues Met1-Leu5 – red, IL-1RII Asp315 C^{α} – magenta.

Table S1. Binding data of heme-incubated IL-36α, trIL-36α, trIL-36β, trIL-36γ as well as of IL-36α protein mutants. SPR, UV/Vis, and resonance Raman spectroscopy were applied to the individual heme-incubated protein variants as required.

Protein	SPR K_{D1} [µM]	SPR K_{D2} [µM]	UV/Vis nUV K_{D1} [µM]	UV/Vis Soret K_{D2} [µM]	Raman coordination state
trIL-36α*	12.7<br>9.3	3.0<br>4.4	n.d.	n.d.	n.d.
trIL-36β*	10.0<br>13.9	4.1<br>3.7	n.d.	n.d.	n.d.
trIL-36γ*	8.9<br>15.9	5.3<br>4.1	n.d.	n.d.	n.d.
IL-36α	6.1<br>4.4	10.2<br>7.1	2.69 ± 1.15<br>~369 nm	11.50 ± 3.06<br>~416 nm	5c
Y108S	n.d.	n.d.	-	30.69 ± 7.49<br>~418 nm	5c
H109A	n.d.	n.d.	0.77 ± 0.53<br>~369 nm	10.35 ± 3.16<br>416 nm	5c
Y108SH109A	n.d.	n.d.	n.p.*<br>~368 nm	13.64 ± 4.69<br>~420 nm	5c
C136SP137A	n.d.	n.d.	-	12.69 ± 4.43<br>~415 nm	5c**
Y108SC136SP137A	n.d.	n.d.	-	15.17 ± 4.35<br>~419 nm	5c
H109AC136SP137A	n.d.	n.d.	-	13.29 ± 3.12<br>~417 nm	5c
Y108SH109AC136SP137A	n.d.	n.d.	-	14.23 ± 3.82<br>~421 nm	5c**

K_D: dissociation constant, nUV: near UV, n.d.: not determined, n.p.: not possible, *Proteins for SPR studies were purchased from R&D systems (see materials). **Negligible indication of hexacoordination.

Table S2. Potential HRMs in IL-36α, IL-36β, and IL-36γ based on cysteine, histidine, or tyrosine as heme-coordination site. Probability for heme binding was evaluated based on the sequence composition, the location within the protein structure, and the net charge of the potential sequence stretches (Brewitz et al., 2015, 2016). Protein sequences were retrieved from Uniprot (Bateman et al., 2017).

No.	Position	Amino acid sequence	Net charge	Heme-binding potential
		**IL-36α**		
**m1**	H20	QDIN**H**RVWV	0/(+1)*	-
**m2**	C49	ALIS**C**R**H**VE	0/(+1)*	-
**m3**	H51	IS**C**R**H**VETL	0/(+1)*	-
**m4**	Y64	GNPI**Y**LGLN	0	-
**m5**	C73	GLNL**C**LM**C**A	0**	-
**m6**	C76	L**C**LM**C**AKVG	+1**	-
**m7**	Y96	IMDL**Y**NQPE	-2	-
**m8**	Y108	SFLF**YH**SQS	0/+1	+
**m9**	H109	FLF**YH**SQSG	0/+1	+
**m10**	C136	SEGG**CP**LIL	-1	+
		**IL-36β** (isoform 1)		
**m1**	Y12	APKS**Y**AIRD	+1	+
**m2**	H44	PVTL**H**LIA**C**	0/(+1)*	-
**m3**	C48	**H**LIA**C**RDTE	-1/(+0)*	-
**m4**	Y63	GNMV**Y**LGIK	+1	-
**m5**	C72	GKDL**C**LF**C**A	0	-
**m6**	C75	L**C**LF**C**AEIQ	-1	-
**m7**	C100	GKDT**C**WKLV	+1	+
**m8**	H107	LVGI**H**T**C**IN	0/(+1)*	-
**m9**	C109	GI**H**T**C**INLD	-1/(+0)*	-
**m10**	C118	VRES**C**FMGT	0	-
**m11**	H141	SSFQ**HHH**LR	+1/(+4)*	+
**m12**	H142	SFQ**HHH**LRK	+2/(+5)*	+
**m13**	H143	FQ**HHH**LRKK	+3/(+6)*	+
		**IL-36β** (isoform 2)		
		m1-m6 are identical to isoform 1 of IL-36β.		
**m7**	Y95	IMDL**Y**VEKK	0	-
**m8**	H108	FLFF**H**NKEG	0	+
**m9**	Y123	QSVS**Y**PGWF	0	-

**m10**	Y153	NTNF**Y**LDSV	-1	-
		**IL-36γ** (isoform 1)		
**m1**	Y16***	GRAV**Y**QSM**C**	+1	+
**m2**	C20	**Y**QSM**C**KPIT	+1	+
**m3**	C61	AVIT**C**K**Y**PE	0	-
**m4**	Y63	IT**C**K**Y**PEAL	0	-
**m5**	Y76	GDPI**Y**LGIQ	-1	-
**m6**	C85	NPEM**C**L**YC**E	-2	-
**m7**	Y87	EM**C**L**YC**EKV	-1	-
**m8**	C88	M**C**L**YC**EKVG	0	-
**m9**	Y108	IMDL**Y**GQPE	-2	-
**m10**	Y120	PFLF**Y**RAKT	+2	-
**m11**	Y159	LGKS**Y**NTAF	+1	+
		**IL-36γ** (isoform 2)		
**m1**	Y16***	GRAV**Y**QSIT	+1	+
**m2**	C26	AVIT**C**K**Y**PE	+1	+
**m3**	Y28	IT**C**K**Y**PEAL	0	-
**m4**	Y41	GDPI**Y**LGIQ	0	-
**m5**	C50	NPEM**C**L**YC**	-1	-
**m6**	Y52	NPEM**C**L**YC**E	-2	-
**m7**	C53	EM**C**L**YC**EKV	-1	-
**m8**	Y73	M**C**L**YC**EKVG	0	-
**m9**	Y85	IMDL**Y**GQPE	-2	-
**m10**	Y124	PFLF**Y**RAKT	+2	-
**m11**	Y164	LGKS**Y**NTAF	+1	+
		**IL-36Ra**		
**m1**	C8	SGAL**C**FRMK	+2	+
**m2**	Y20	LKVL**Y**L**H**NN	+1(+2*)	+
**m3**	H22	VL**Y**L**H**NNQL	0/(+1*)	+
**m4**	H32	AGGL**H**AGKV	+1	-
**m5**	C67	GGSQ**C**LS**C**G	0	-
**m6**	C70	Q**C**LS**C**GVGQ	0	-
**m7**	Y89	IMEL**Y**LGAK	0	-
**m8**	Y101	SFTF**Y**RRDM	+1	-
**m9**	Y116	ESAA**Y**PGWF	-1	-
**m10**	C122	GWFL**C**TVPEA	-1	-
**m11**	Y150	ITDF**Y**FQQ**C**	-1	-
**m12**	C154	**Y**FQQ**C**D	-1	-

**m13**	Y164	LGKS**Y**NTAF	+1	+

*Neutral charge if H involved as coordination site and is not considered as additional basic residue. **Cysteines alone were shown to be much less or ineffective for heme binding(Brewitz et al., 2015). ***Will be removed upon proteolytic truncation to achieve biological activity.

Table S3. Synthesized peptides and respective peptide mutants of potential HRMs derived from IL-36α. Peptides were synthesized via standard Fmoc-based solid phase peptide synthesis and subsequently analyzed by MS, HPLC, and TLC.

No.	Peptide sequence	M_w[a] (M_w theor.) [g/mol]	Preparative HPLC t_r [min]	Analytical HPLC t_r [min]	TLC R_f
**1**	FLFYHSQSG	542.77[a] (1083.51)	71.9[c]	20.4[e]	0.57[g]<br>0.71[h]
**2**	SEGGAPLIL	855.49[b] (854.49)	65.3[d]	20.6[f]	0.66[g]<br>0.73[h]
**3**	SEGGCALIL	861.43[b] (860.43)	62.9[d]	21.5[f]	0.57[g]<br>0.71[h]
**4**	SEGGAALIL	829.47[b] (828.47)	59.7[d]	20.5[f]	0.66[g]<br>0.73[h]
**5**	FLFYASQSG	1018.49[b] (1017.49)	83.3[c]	21.2[e]	0.66[g]<br>0.88[h]
**6**	FLFAHSQSG	496.75[a] (991.49)	66.4[c]	17.8[e]	0.51[g]<br>0.69[h]
**7**	FLFAASQSG	463.74[a] (925.47)	75.5[c]	19.2 [e]	0.61[g]<br>0.85[h]

Peptides were measured with LC-ESI-MS and peaks were detected as [a]$[M+2H]^{2+}$ or $[M+H]^{+}$. Semi-preparative RP-HPLC was applied for peptide purification using [c]0-50% eluent B (0.1% TFA in 90% acetonitrile, eluent A: 0.1% TFA in water) in 120 min and [d]10-60% eluent B in 120 min as gradients. Analytical HPLC was performed using [e]10-50% eluent B (0.1% TFA in acetonitrile, eluent A: 0.1% TFA in water) in 40 min and [f]10-40% eluent B in 30 min. For TLC the following systems were applied: [g]n-butanol/acetic acid/water (48:18:24, v/v) and [h]pyridine/ethyl acetate/acetic acid/water (5:5:1:3, v/v).

Table S4. Data of UV/Vis- and resonance Raman spectroscopy of IL-36α-derived peptides and respective mutants (**1-7**) in complex with heme. (For peptide sequences see Table S3.)

No.	K_D for UV/Vis [µM] (wavelength)	Raman coordination state
**1**	4.48 ± 2.20 (~415 nm)	5c(6c)
**2**	n.b.	-
**3**	n.d. (~319 nm, ~416 nm)	5c
**4**	n.b.	-
**5**	2.29 ± 1.32 (~418 nm)	5c
**6**	n.b.	-
**7**	n.b.	n.p.

K_D, dissociation constant; n.b. no binding; n.p., not possible; 5c, pentacoordinated; 6c, hexacoordinated.

Table S5. Structural statistics of the refined NMR solution structure of IL-36α.

**Total distance restraints**	4107
• intra (|i-j|=0)	350
• sequential / short (|i-j|=1)	1158
• medium (1<|i-j|<5)	767
• long range (|i-j|≥5)	1832
hydrogen bond	72
**Target function** [$Å^2$]	
after (before) energy minimization	4.13 (4.14)
**AMBER physical energies** [$kcal/mol^{-1}$]	
before energy minimization	-4526 ± 97
after energy minimization	-5812 ± 101
**R.M.S.D.** [Å]	
mean global r.m.s.d. values	
all heavy atom (residues 10-158)	1.52 ± 0.21
backbone (residues 10-158)	0.74 ± 0.12
**Ramachandran plot** [%]	
Residues in most favored regions	69.3
in additionally allowed regions	28.7
in generously allowed regions	1.9
in disallowed regions	0.1

Table S6. CD spectroscopy analysis of the secondary structure content of IL-36α and trIL-36α (6-158).

Protein	Analysis tool	α-helix	β-sheet	irregular
IL-36α	CAPITO	10-13%	30-35%	50-60%
IL-36α	K2D3	9.8%	19.7%	70.5%
trIL-36α	CAPITO	10-13%	32-37%	51-59%
trIL-36α	K2D3	8.5%	19.5%	72.0%

Table S7. Backbone RMSD based structural assessment of wild-type IL-36α and its mutants in a 200 ns MD simulation.

**Protein**	**RMSD against model 1 of the NMR ensemble (Å)**	**RMSD against structure from MD equilibration (Å)**
IL-36α	3.34 ± 0.52	2.516 ± 0.6
Y108S	3.06 ± 0.32	2.32 ± 0.56
H109A	3.11 ± 0.24	2.04 ± 0.65
Y108SH109A	2.75 ± 0.54	3.00 ± 1.18
C136SP137A	2.83 ± 0.61	2.63 ± 0.46
Y108SC136SP137A	3.17 ± 0.53	2.54 ± 0.82
H109AC136SP137A	3.67 ± 0.31	2.16 ± 0.5
Y108SH109AC136SP137A	3.30 ± 0.52	1.89 ± 0.67

RMSD of reference structure 1 w.r.t. NMR ensemble = 0.84 Å;

Movie Information

Movie S1: Heme binding to both C136 and Y108 residues on the surface of the IL-36α wild-type protein. The video is a screen recording of a fast-forwarded molecular dynamics trajectory. The video shows a simulation cell containing the IL-36α protein with two heme molecules bound to its surface as a result of molecular docking simulations. The protein is displayed in the cartoon style, colored gray. On the protein structure, the residues Y108, H109, and C136 are shown as sticks colored green, magenta, and yellow, respectively. Although the H109 residue does not participate in heme coordination, its orientation is relative to the Y108 residue. The heme molecule bound to the Y108 site is shown as red spheres, while the one bound to the C136 residue is shown as blue spheres. It can be clearly observed that both heme molecules stay bound to the surface of the protein.

Movie S2: Heme binding to the Y108 residue on the surface of the C136SP137A mutant of IL-36α. The video is a screen recording of a fast-forwarded molecular dynamics trajectory. The video shows simulation cell containing the C136SP137A mutant of IL-36α protein with a heme molecule bound to its surface as a result of molecular docking simulations. The protein is displayed in the cartoon style, colored gray. On the protein structure, the residues Y108 and H109 are shown as sticks colored green and magenta, respectively. Although the H109 residue does not participate in heme coordination, its orientation is relative to the Y108 residue. The heme molecule bound to the Y108 site is shown as red. It can be clearly observed that the heme molecule is quickly dislodged from its binding site on the Y108 residue and moves into the solution shortly after the simulation start. This video shows that in the absence heme binding to the C136 residue heme binding to the Y108 residue is short-lived.
